# Impaired Insulin Signaling Alters Mediators of Hippocampal Synaptic Dynamics/Plasticity: A Possible Mechanism of Hyperglycemia-Induced Cognitive Impairment

**DOI:** 10.3390/cells12131728

**Published:** 2023-06-27

**Authors:** Mubeen A. Ansari, Aishah Al-Jarallah, Fawzi A. Babiker

**Affiliations:** 1 Department of Pharmacology and Toxicology, Faculty of Medicine, Kuwait University, Kuwait City 13110, Kuwait; 2 Department of Biochemistry, Faculty of Medicine, Kuwait University, Kuwait City 13110, Kuwait; 3 Department of Physiology, Faculty of Medicine, Kuwait University, Kuwait City 13110, Kuwait

**Keywords:** streptozotocin, synapse, acetylcholine esterase, Na^+^/K^+^-ATPase, cognitive behavior

## Abstract

**Simple Summary:**

An early time course study of the proteins associated to synaptic dynamics and cognitive behavior. Study evaluated the expression levels and activation state of markers in hippocampal synaptosomes. Changes in enzymatic activities and proteins levels were detected early during the disease. Study used two different models of impaired insulin signaling in the brain. The two models used exhibited different effects on the parameters tested.

**Abstracts:**

In hyperglycemia/diabetes, impaired insulin signaling in the brain causes a cerebral pathology and cognitive impairments as in sporadic Alzheimer’s disease (sAD). To improve our understanding of the role of impaired insulin signaling in cognitive dysfunction, we examined the expression levels and activation states of mediators of neuronal survival and cytoskeletal dynamics/plasticity in the hippocampus in rats treated with intraperitoneal (IP) and intracerebroventricular (ICV) streptozotocin (STZ). We studied early changes (1, 3, or 6 weeks) in response to STZ treatment. Cognitive function was assessed using the novel object recognition (NOR) test and T-maze. The activity of acetylcholine esterase (AChE) and Na^+^/K^+^-ATPase was measured biochemically and the expression levels and phosphorylation states of mediators of neuronal survival and synaptosomal dynamics/plasticity (PI3K, Akt, GSK-3β, PAK, LIMK-1, and cofilin-1) were examined by immunoblotting. Significant cognitive losses were observed in STZ-injected rats, together with elevated AChE and reduced Na^+^/K^+^-ATPase activity. STZ administration reduced the ratio of phosphorylated/total PI3K, Akt, and GSK-3β and increased the ratio of phosphorylated/total PAK, LIMK-1, and coflin-1. Significant differences between the IP and ICV administration of STZ were observed in the expression levels and phosphorylation states of different markers. Cognitive impairments at 3W and 6W were significantly correlated with changes in the ratio of phosphorylated/total proteins. We conclude that impaired insulin signaling causes early deleterious changes in mediators of synaptic dynamics/plasticity. This study provides mechanistic insights into early events involved in mediating sAD and highlights potential intervention opportunities.

## 1. Introduction

Alzheimer’s disease (AD) is a neurological condition that affects the elderly and is characterized by progressive and irreversible neurodegeneration in the cerebral cortex. It is viewed as a cognitive loss (dementia), resulting in the patient’s loss of self-sufficiency. In adults (<65 years), the development of dementia is uncommon and is termed sporadic AD (sAD). Excluding genetic abnormalities in familial AD (fAD), various random risk factors are associated with the development/progression of sAD [1,2]. A growing body of evidence indicates that the development of sAD could indirectly be associated with hyperglycemia/diabetes or decreased insulin function in the brain. Impaired insulin signaling in the brain contributes to synaptic dysfunction and cognitive deficits, thus linking hyperglycemia to the development/progression of sAD. Impaired insulin signaling causes oxidative stress [3] and chronic inflammation in the brain, which exacerbate insulin resistance and tissue deterioration and result in an AD-like pathology [1,2,4]. Animal models of streptozotocin (STZ)-induced hyperglycemia/diabetes or neurotoxicity mimic several histopathological hallmarks of AD, including oxidative stress, inflammation, extracellular amyloid-β (Aβ) accumulation, increased intracellular tau protein phosphorylation (p-tau), and loss of cognitive behavior [2,4,5,6,7,8]. STZ-induced insulin resistance and/or impaired insulin signaling in the brain [9] is accompanied by reduced energy metabolism and the attenuated production of adenosine triphosphate (ATP), acetyl-CoA, and acetylcholine (ACh). In addition, simultaneous inflammation induces the production of enormous reactive oxygen and/or nitrogen species (ROS/RNS). Together, oxidative stress and inflammation have been implicated in mediating the damage to myelinated tracts, synaptic contacts, and hippocampal neuronal circuits, which may explain the cognitive impairments observed in animal models of diabetes and impaired insulin signaling] [3,10,11,12]. 

The pathology of hyperglycemia/diabetes progresses over time. The intraperitoneal (IP) injection of STZ (~60 mg/kg) in rats causes hyperglycemia within one week; however, the development of other symptoms, including damage to the central nervous system (CNS) [13] and peripheral nervous system (PNS) [14], appears later in the course of the disease. In the early stages, hyperglycemia affects different neuronal pathways [15,16], including the cholinergic system in the celiac ganglia (impairs glucagon response to insulin) [17]. Hyperglycemia reduces the expression of genes that regulate hippocampal synaptic plasticity, including histone deacetylases and glycogen synthase kinase-3β (GSK3β) [18], and neuronal long-term potentiation (LTP) [19]. Hyperglycemia/diabetes and sAD increase ACh esterase (AChE) activity and alter phosphatidylinositol-3 kinase (PI3K)/protein kinase B (Akt)/GSK3β-mediated signaling in the cerebral cortex [20,21]. In addition, oxidative stress and inflammation modify PI3K/Akt/GSK3β-mediated signaling and increase tau protein phosphorylation [20,21]. In chronic conditions, these effects are aggravated by increased synaptosomal lipid peroxidation [22] and reduced α-amino-3-hydroxy-5-methyl-4-isoxazolepropionic acid (AMPA) binding to its receptor (glutamate receptor-1 (GluR1)), prominently in the hippocampus [23]. Moreover, hyperglycemia/diabetes attenuates Na^+^/K^+^-ATPase activity and LTP in the hippocampus [24] and disrupts synaptic potentiation [25], resulting in the progressive loss of cognitive behavior in diabetic animals [26]. 

Similarly, but more profoundly impaired insulin signaling in the brain was observed when rodents received direct intracerebroventricular (ICV) injections of STZ (3.0 mg/kg) [27,28,29]. Cognitive impairments were observed two weeks post-ICV-STZ injection. In addition, a marked increase in inflammation, oxidative stress, and apoptotic cell death [30] aggravated the cognitive loss over time through the attenuated expression of insulin receptors (IRs) and the phosphorylation of PI3K/Akt/GSK-3β (p-PI3K/p-Akt/p-GSK-3β) in the cerebral cortex [31]. Reduced hippocampal LTP [32], the increased accumulation of phosphorylated tau [2,33], and neuronal dysfunction [29,33,34] were also reported. Animals exhibited augmented neuroinflammation [4,35], oxidative stress [3,36], enhanced AChE activity [10,11,37], and cognitive deficits [38] over time post-ICV-STZ administration. 

The loss of dendritic spines prior to neuronal death in AD was reported to be due to dysregulation in the function of actin-depolarizing factors cofilin-1 (the major neuronal component; n-cofilin) [39] and cofilin-2 (muscle; m-cofilin). The phosphorylation and dephosphorylation of cofilins are mediated by p21-activated kinase (PAK)/Lin-11/Isl-1/Mec-3 kinase (LIMK-1/2) and slingshot (SSH1) proteins, respectively [40,41,42,43,44]. Active (non-phosphorylated) cofilin-1 interacts with PI3K and PAK [45,46] and regulates synaptic dynamics/plasticity in dendritic spines [47,48,49]. Dendritic spine density/function declines with aging and in patients with neurological disorders [50,51,52] and in experimental models of the disease [53,54,55]. Increased expression of LIMK and p-cofilin-2 in patients with metabolic syndrome and diabetes [56,57] supports the role of impaired insulin signaling in cytoskeletal/synaptic structures. These changes were reported in vitro and in vivo [58,59,60]. IP injections of STZ disrupted the blood–brain barrier [61], decreased dendritic spines, augmented LIMK-1 and cofilin-1 phosphorylation in the hippocampus, and impaired cognitive function [62]. Furthermore, ICV injection of STZ resulted in the loss of cognition, synaptic degeneration, and increased hippocampal LIMK-1/cofilin-1 phosphorylation [63,64,65,66]. Altered hippocampal LIMK/cofilin signaling resulted in synaptic changes in the cerebral cortex [63,64,65,66]. 

In this study, we aimed to investigate the involvement of early-time-course changes in proteins mediating synaptic dynamics/plasticity, specifically the PI3K and PAK pathways, in response to impaired insulin signaling. Our data indicate early changes in the expression levels and activation states of the PI3K/Akt/GSK-β and PAK/LIMK-1/coflin-1 pathways in response to the IP and ICV administration of STZ. The ICV route, however, resulted in more profound changes in the expression levels and activation of PI3K, Akt, and PAK relative to the IP route. STZ-induced changes in these pathways correlated with the impaired cognitive function observed in hyperglycemic/diabetic rats. We conclude that impaired insulin signaling results in early changes in mediators of neuronal survival and synaptic dynamics/plasticity. Taken together, our findings suggest that impaired insulin signaling triggers early changes in the mediators of neuronal survival and synaptic dynamics/plasticity, which may play a key role in the progression of sAD.

## 2. Materials and Methods

### 2.1. Chemicals

Pierce^®^ BCA protein assay reagents and protease inhibitor mini tablets were purchased from Thermo Fisher Scientific, Inc. (Pittsburgh PA, USA). Materials applied in electrophoresis were purchased from Bio-Rad Laboratories, Inc. (Hercules CA, USA). Mouse monoclonal anti-PI3K (sc-166365), Akt (sc-81434), p-Akt (sc-514032), detecting isoforms 1/2/3, GSK-3β (sc-53931), p-GSK-3β (sc-373800), PAK (sc-166174), detecting α/β/γ isoforms, LIMK-1 (sc-28370), cofilin-1 (sc-53934), p-cofilin-1 (sc-271921), and β-actin (sc-47778) antibodies were purchased from Santa Cruz Biotechnology (Santa Cruz, CA, USA). Rabbit polyclonal antibodies of p-PI3K (ab182651) and p-PAK (ab40795) were purchased from abcam (Boston, MA, USA) and p-LIMK-1 (PA5-118698) from Thermo Fisher Scientific (Waltham, MA, USA). Alkaline-phosphatase-conjugated secondary antibodies (anti-mouse (A3562) and anti-rabbit (A3687)) and all other chemicals/reagents, including STZ (2-deoxy-2-(3-[methyl-3-nitrosoureido]-d-glucopyranose)), were purchased from Sigma (St. Louis, MO, USA) unless stated otherwise. 

### 2.2. Animals

In-house-bred three-month-old adult male Wistar rats (365–400 g) of the same batch of breeding were used in this study. All rats were generously provided and maintained during experiments by the Animal Resources Center (ARC), Health Science Center, Kuwait University. Food and water were available ad libitum for the animals. The animals were housed in a room at the ARC, which was maintained under controlled conditions (maintained at 25 ± 2 °C temperature, with 50% relative humidity and 12-h light/dark cycle). The Health Sciences Center Animal Research Ethics Committee, Kuwait University, which follows the recommendations of the NIH Guidelines for the Care and Use of Laboratory Animals, approved the surgical procedures and post-operative maintenance implemented in the study. 

### 2.3. Animal Groups and Experimental Design

Rats injected with intraperitoneal (IP) STZ (IP-STZ) and intracerebroventricular (ICV) STZ (ICV-STZ) were used to assess the effect of time on cognitive function and changes in hippocampal synaptosomal proteins, following our recent experimental design published in [3]. The study was performed blindly; animals were randomized and were assessed by different investigators. One hundred and twenty rats were divided into 12 groups (*n* = 10/group), six groups for IP treatment and six for ICV injection. Group (i) IP vehicle control—one week (IP-Veh-1W); (ii) IP-STZ—one week (IP-STZ-1W); (iii) IP vehicle control—three-weeks (IP-Veh-3W); (iv) IP-STZ—three weeks (IP-STZ-3W); (v) IP vehicle control—six weeks (IP-Veh-6W); and (vi) IP-STZ—six weeks (IP-STZ-6W). Rats in the IP-Veh groups received IP injection of 0.5 mL of vehicle (citrate buffer (0.1M) pH 4.6) and rats in the IP-STZ groups received 55 mg/kg STZ, in 0.5 mL of 0.1 M citrate buffer, once (on day 1). In IP-STZ-treated rats, the blood glucose level on day 4 (and monitored every 7th day) was measured. Blood glucose 17.5 mmol/L was used as a cut-off value for hyperglycemia/diabetes. Group (vii) ICV vehicle control—one-week (ICV-Veh-1W); (viii) ICV-STZ—one-week (ICV-STZ-1W); (ix) ICV vehicle control—three weeks (ICV-Veh-3W); (x) ICV-STZ—three weeks (ICV-STZ-3W); (xi) ICV vehicle control—six-weeks (ICV-Veh-6W), and (xii) ICV-STZ—six weeks (ICV-STZ-6W). In the ICV model, control rats received a single injection of 5 µL vehicle (sterile citrate buffer (0.1 M, pH 4.6) prepared in artificial cerebral spinal fluid (CSF)) and STZ-treated rats received 5 µL STZ solution (3.0 mg/kg) prepared in the vehicle on day 1, administered into both the left and right lateral ventricles. This dose was selected based on our previous studies [3,10,11]. The solution was administered slowly into the lateral ventricle (over a period of 20 min) to allow reasonable diffusion in the cerebral tissue. In ICV-STZ-injected rats, blood glucose levels were also monitored on every 7th day, and none of these rats showed a marked increase in blood glucose. Rats from the 3W and 6W groups (of the IP and ICV models) were assessed for cognitive performance (working memory) in the T-maze and novel object recognition (NOR) tests. Rats were sacrificed (euthanized with CO_2_ and then decapitated) at 1W, 3W, and 6W; their brains were extracted; and the left and right hippocampi were dissected, snap-frozen in liquid nitrogen, and stored in a deep freezer at –80 ^°^C. 

### 2.4. Surgical Procedure for ICV- STZ/Vehicle Injection

During the surgical procedure, aseptic conditions were maintained to eliminate the risk of infection in the animals following surgery. Vehicle or STZ injections into the lateral ventricles were performed using our previously described protocol [3,10,11]. Surgery kits, tools, and materials were autoclaved, and the workstation, including the stereotaxic apparatus, was sterilized with 70% ethanol. Ten minutes prior to the surgical procedure, each rat was anesthetized with an intramuscular injection of ketamine/xylazine hydrochloride solution (ketamine 60 mg/kg, xylazine 5 mg/kg). The rat was placed on a stereotaxic apparatus, fixed with the help of ear bars. The skullcap was exposed via a small skin incision and two points were marked for drilling a bur hole on either side of the midline (1.5 mm from midline), 0.8 mm posterior to the bregma. Both holes were drilled carefully up to the level of the dura mater, without damaging any brain tissue. Through these holes, a 10 µL Hamilton syringe (of a 26 G needle) filled with the vehicle (citrate buffer) or STZ was inserted at a 4.0 mm depth (measured from the level of the dura mater) to reach the lateral ventricle. In control groups, 5.0 µL vehicle (sterile citrate buffer (0.1M, pH 4.6) in artificial CSF) was injected slowly (over a period of 20 min) into both the left and right lateral ventricles once on day 1 of the experiment. ICV-STZ groups were injected with 5.0 µL STZ solution (1.5 mg/kg/ventricle) prepared in the vehicle. Before withdrawal, the needle was kept in place for an additional five minutes to avoid the backflow of the injected solutions. Then, the holes were sealed with dental acrylic, the skin wound was closed with sutures, and an antiseptic betadine solution was applied on the wounded skin. After the surgical procedure, rats were kept in a warm area until they had recovered from the anesthesia. Rats were housed individually until the end of the experiment. Antiseptic betadine solution was applied daily on the wounded area for three days post-surgery and wet food was offered.

### 2.5. Cognitive Behavior Tests

#### 2.5.1. Novel Object Recognition (NOR) Test

Impairments of emotional memory (fear conditioning and sensorimotor gaiting) and non-emotional memory (novel object recognition (NOR) and different maze tests) have been used to assess cognition in experimental models of sAD disease [67,68,69,70,71]. The NOR test is a working memory task (exploration) that depends on the rat’s innate tendency to investigate novel compared to familiar objects. This test relies on the function of the cerebral cortex and the hippocampus, to a lesser extent. It assesses non-spatial memory tasks in rats. The setup consists of an open arena (open box of 60 cm × 60 cm × 50 cm), a video recording system, and three plastic objects (two identical and one different). The NOR test that we used in this study followed a standard procedure, as described in [72,73], with some modifications [74,75]. Initially, rats were placed into the box without any object on 3 consecutive days, for 2 min per day. On the 4th day, each rat performed two trials (T1 and T2). T1 was conducted in the presence of two identical objects (placed in the right and left corners) in the testing box for 10 min, allowing exploration and familiarization. This was followed by a 1-h inter-trial interval, during which the testing box was cleaned with 70% isopropanol and rats were returned to their home cages. T2 was performed with a familiar object and a new object for 5 min. Exploration behavior was defined as the movement of the rat toward the object within 2 cm (marked around the object) and/or touching the object. The object exploration time (sec) was recorded with a stopwatch. The discrimination ratio (DR) = [TN/(TF + TN); TF = time spent exploring familiar object and TN = time spent exploring novel object] was calculated to evaluate the recognition memory of the individual rats. The values reflecting the exploration of the novel object were then converted into the percentages of change versus vehicle-treated rats (% of control).

#### 2.5.2. T-Maze Test

The T-maze test is a well-known task used to study rodents’ working and spatial memory. The test relies on the function of the medial temporal lobe, including the hippocampus, and requires the recall of the previous task using a “place” strategy. It can be performed in two manners, either free or forced tests, using a “T”-shaped design [76,77,78]. Various protocols are described in the literature that vary in terms of the number of trials, type of reward, length of experiment, and methodology used [78]. In our study, we utilized a black plastic T-maze (60 × 20 × 30 cm stem and 45 × 20 × 30 cm each arm) and an appetite reward task. In this task, the animal learns to find the baited arm based on their memory retrieval ability regarding the previously visited arm and chooses the opposite arm to obtain a food reward. We performed the T-maze test as previously described in [76,79], with some modifications. Briefly, two days prior to the experiment, rats were deprived of ad libitum food (85%). First, rats were habituated to the apparatus for one day; then, they were trained against their preference in the following training trials (10 trials/day for 5 days) by baiting the alternate arm of the maze. During the trial, each rat was placed in the stem arm of the T-maze and allowed to select a goal arm to enter. Once the rat chose a goal arm, it was restrained to this arm for 30 s before returning to the stem arm. The rat’s entry into the baited arm was scored as a correct choice, while entry into the unbaited arm was scored as an incorrect choice. The T-maze was cleaned with 70% isopropanol before starting the trial of the next animal, to eliminate odors. To assess working memory, rats were subjected to an additional probe trial (on day 6), 1 h after the last training trial, with the maze being rotated 180°. However, all cues (including the experimenter) remained in the same positions. The average reduction in the number of incorrect choices (errors) per day for 5 days of training was recorded as the rat’s working memory. During the probe test, a correct choice was designated as working memory, while an incorrect choice (making the same body turn as in training trials) was designated as a spontaneous response strategy. The number of incorrect choices during training sessions was converted into percentage values for the groups (% of errors). The number of correct choices during the probe test was converted into a percentage (% of correct choice) versus vehicle-treated rats. The number of incorrect choices (% of errors) during the probe test was used for the analysis of the correlation with changes in synaptic markers.

### 2.6. Isolation of Hippocampal Synaptosomal Fractions

Hippocampal tissue was dissected and synaptosomal fractions were prepared as described in our previous study [3], with some modifications. Briefly, hippocampal tissue was homogenized in chilled sucrose (250 mmol/L) isolation buffer (pH 7.2) containing 1.0 mmol/L EDTA, 10 mmol/L HEPES, and protease inhibitors (4.0 Kg/mL leupeptin, 4.0 Kg/mL pepstatin, 5.0 Kg/mL aprotinin, 20.0 Kg/mL trypsin inhibitor). The homogenates were centrifuged at 1350× *g* at 4 °C for 5 min. The supernatants were again centrifuged at 21,200× *g* at 4 °C for 10 min and the pellets were collected and resuspended in isolation buffer. The solution was layered over a discontinuous sucrose gradient (1.0 M, pH 8.0 and 1.18 M, pH 8.5) spun at 85,500× *g* at 4 °C for 1 h. Synaptosomal fractions were then collected from the interface, resuspended in isolation buffer, and spun at 32,000× *g* at 4 °C for 20 min. Isolated synaptosomal fractions from each individual rat hippocampus were used for the biochemical analysis.

### 2.7. Estimation of Protein

The total protein concentration in each isolated synaptosomal fraction was determined using Pierce^®^ BCA protein assay reagents (Pittsburgh PA, USA).

### 2.8. Estimation of Acetylcholinesterase (AChE) and Na^+^/K^+^-ATPase Activity

The activity of AChE (EC 3.1.1.7) in isolated synaptosomes was measured using the method of Ellman et al. (Ellman GL et al., 1961), as described previously [10], with some modifications. Briefly, phosphate buffer (0.1 M, pH 8.0), 1.0 mM acetylthiocholine iodide, and buffered Ellman’s reagent (DTNB 10.0 mM, NaHCO3 15.0 mM) were mixed and incubated for 5 min at room temperature. Then, 180 µL of the reaction mixture was added to 20 µL of the synaptosomal sample in a 96-well plate. The changes in absorbance were recorded at 412 nm for 5 min and AChE activity was calculated as nmol thiocholine formed min^−1^ mg^− 1^ protein, using a molar extinction coefficient of 13.6 × 10^3^ M^−1^ cm^−1^.

The activity of Na^+^/K^+^-ATPase (EC 3.6.1.37) was measured as inorganic phosphorus (Pi) release using the method of Sovoboda and Mossinger [80], with slight modifications, as described previously [11]. The activity of Na^+^/K^+^-ATPase was determined in two reaction mixtures, A and B. A total of a 1.0 mL volume of reaction mixture A (0.2 M KCl, 1.0 M NaCl, 0.1 M MgCl2, 0.2 M Tris–HCl buffer (pH 7.4), and 25 mM ATP) and 50 µL of the synaptosomal sample was prepared. In another set, a total volume of 1.0 mL of reaction mixture B (0.1 M MgCl2, 10 mM ouabain, 1.0 M NaCl, 0.2 M Tris–HCl buffer (pH 7.4), and 25 mM ATP) and 50 µL of the synaptosomal sample was prepared. Reaction mixtures were incubated at 37 °C for 15 min and the reaction was stopped by adding 750 µL of chilled 10% trichloroacetic acid (TCA). Thereafter, reaction mixtures were centrifuged 5000× *g* at 4 °C for 10 min. Collected supernatants (250 µL of each sample) were used for the estimation of inorganic phosphorous, calculated as nmol Pi formed min^−1^ mg^−1^ protein. The values of AChE and Na^+^/K^+^-ATPase activity were then converted into percentages of change versus vehicle-treated rats (% of control).

### 2.9. Western Blot Analysis for Proteins of Synaptic Dynamics/Plasticity

The expression levels of proteins involved in neuronal survival and synaptic dynamics/plasticity (PI3K, Akt, GSK-3β, PAK, LIMK-1, and cofilin-1) were examined by immunoblotting, as described in our previous studies [81,82], with some modifications. β-actin was used as a loading control. Synaptosomal proteins (20µg) were diluted in 1-X loading buffer (1:1) and loaded on a 4–20% Tris–HCl gradient gel (Bio-Rad) along with a protein ladder. Proteins were transferred to a nitrocellulose membrane using a transfer buffer (25 mM Tris, 150 mM glycine, and 20% MeOH) in a semidry transfer system (Bio-Rad) at 15 V for 2 h. Thereafter, the blots were blocked with 5% fat-free dry milk (for non-phosphorylated proteins) or 3% bovine serum albumin (BSA; for phosphorylated proteins) in Tris-buffered saline with Tween-20 (TBST). Primary antibodies were added at the corresponding concentration of 1:200–1:1000 as per the manufacturer’s instructions, and blots were incubated overnight at 4 °C. After washing them 3 times in TBST for 10 min, the blots were incubated for 1 h with alkaline-phosphatase-conjugated secondary antibodies in a 1:8000 dilution, washed three times in TBST, for 10 min each, and developed using a BCIP/NBT substrate (Sigma Fast tablets). Blots were dried and scanned, and the band density was quantified and expressed as a percentage of change versus vehicle-treated rats (% of control).

### 2.10. Statistical Analysis

Data are expressed as means ± SD. Possible differences between group means (of different variables) were evaluated using one-way analysis of variance (ANOVA) followed by Dunnett’s multiple comparisons. A two-way ANOVA coupled with a Bonferroni post hoc test was applied in comparing the treatment method to time post-injection (treatment x time). Associations among changes in protein expression and activation and cognitive impairments were evaluated using Pearson’s correlation followed by Gaussian distribution (Graph Pad Prism 10, SAS Institute) and expressed in linear regression plots. For significance, alpha was set at 0.05. With the exception of the results of the behavioral experiments, data are presented as the percentage of the respective control group (% of control), along with the F-value, ANOVA significance, and the *p*-values of the comparisons of mean data between selected groups. Analysis of the data revealed no significant differences between the IP-Veh groups of 1W, 3W, and 6W; hence, the mean data of these groups were used to represent the IP vehicle (IP-Veh) in all graphs.

## 3. Results

### 3.1. IP-STZ and ICV-STZ Treatments Attenuated Rats’ Responses in Novel Object Recognition (NOR)

**Figure 1:**
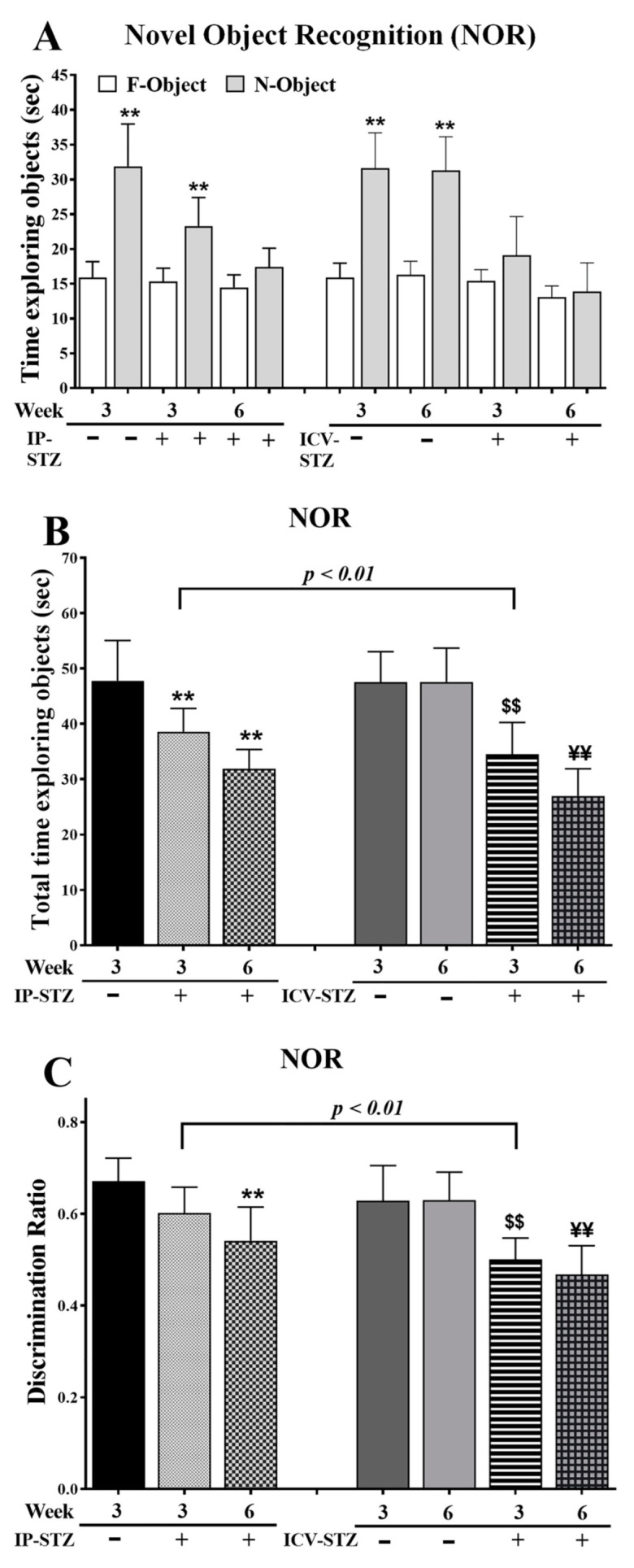
(**A**) Time spent exploring objects (sec), (**B**) total time spent exploring objects (sec) as TF + TN objects, and (**C**) discrimination ratio as DR = TN/(TF + TN) in different groups. Rats with IP-STZ and IVC-STZ injection spent significantly less time exploring novel objects on 4th day of T2 sessions (Veh vs. STZ, ** *p* < 0.01). ICV-STZ-injected rats demonstrated a significant lack of exploration behavior, indicated by the reduced time spent exploring objects and attenuated discrimination ratio at 3W, compared to the IP-STZ group (*p* < 0.01). STZ-injected rats showed a significant reduction in interest/curiosity and memory on 4th day of T2 trials (IP-Veh vs. IP-STZ-3W and -6W, ** *p* < 0.01; ICV-Veh-3W vs. ICV-STZ-3W, $$ *p* < 0.01; ICV-Veh-6W vs. ICV-STZ-6W, ¥¥; *p* < 0.01). Data are mean ± SD of *n* = 10 rats/group.

IP-STZ and ICV-STZ administration reduced the average time spent exploring objects (F = familiar and N = novel), suggesting a lack of interest (Figure 1A). The total time spent exploring F/N objects was significantly different between groups at 3W and 6W post-STZ administration ((7, 63) = 24.59, *p* < 0.0001) (Figure 1B). In addition, the discrimination ratio was significantly reduced in 6W in the IP-STZ group and in 3W and 6W in the ICV-STZ group ((7, 63) = 14.73, *p* < 0.0001) (Figure 1C). The effects of the treatment methods, IP or ICV, on the total time required to explore the object ((3, 63) = 43.93, *p* < 0.009) and the discrimination ratio ((3, 63) = 36.47, *p* < 0.005) were significantly different at 3W but not 6W after STZ injection.

### 3.2. IP-STZ and ICV-STZ Treatments Attenuated Rats’ Cognitive Behavior in the T-Maze Test

**Figure 2:**
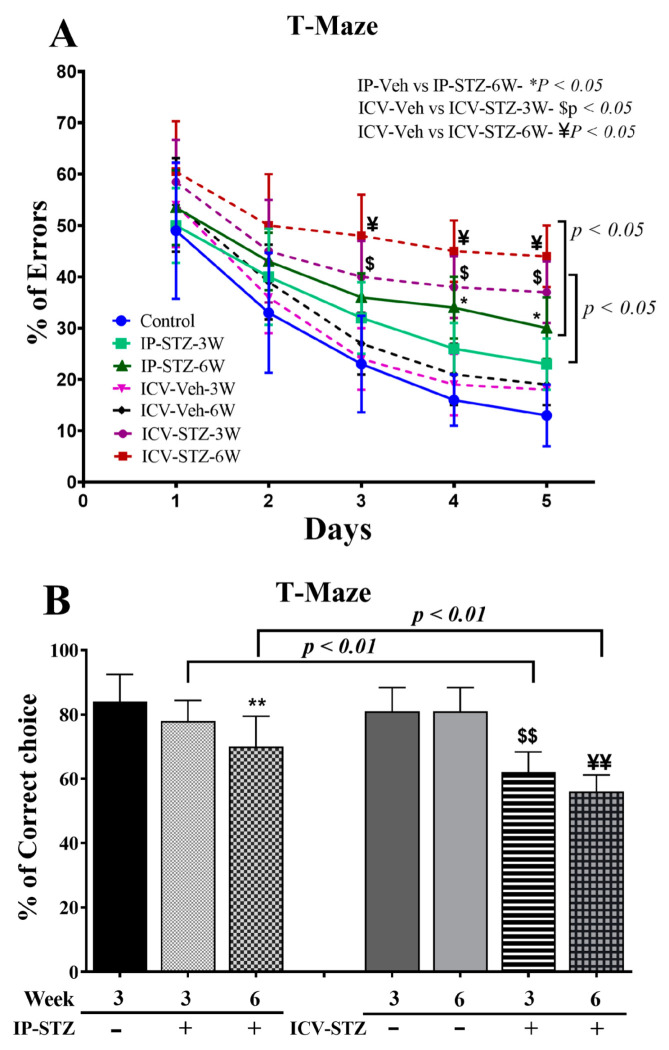
(**A**) Percentage of errors (incorrect responses), (**B**) spontaneous alternative correct choices during probe test in different groups. Rats with IP-STZ and IVC-STZ injection demonstrated significantly higher percentages of incorrect responses on the 3rd, 4th, and 5th days of the learning sessions (IP-Veh vs. IP-STZ-6W, * *p* < 0.05; ICV-Veh-3W vs. ICV-STZ-3W, $ *p* < 0.05; ICV-Veh-6W vs. ICV-STZ-6W, ¥ *p* < 0.05). ICV-STZ-injected rats demonstrated significant learning deficits, indicated by a lower percentage of correct choices and higher percentage of incorrect choices at 3W and 6W, compared to the IP-STZ group (*p* < 0.01). STZ-injected rats showed significant inadequacy in learning and memory on the 5th day, in the probe test (IP-Veh vs. IP-STZ-6W, ** *p* < 0.01; ICV-Veh-3W vs. ICV-STZ-3W, $$ *p* < 0.01; ICV-Veh-6W vs. ICV-STZ-6W, ¥¥ *p* < 0.01). Data are mean ± SD of *n* = 10 rats/group.

IP- and ICV-STZ administration resulted in significant learning deficits, indicated by increased percentages of errors (incorrect responses), in the spontaneous alternative response ((7, 63) = 25.58, *p* < 0.0001) (Figure 2A). Although the percentage error gradually declined over time, the mean numbers of incorrect choices remained significantly higher in IP-STZ (at 6W) and ICV-STZ (at 3W and 6W) rats on days 3, 4, and 5 compared to corresponding vehicle controls (*p* < 0.05). Moreover, the IP and ICV administration of STZ impaired working memory performance, as indicated by the failure to remember the last visited arm of the reward. Working memory function was significantly ((7, 63) = 21.52, *p* < 0.001) affected in both the IP-STZ and ICV-STZ groups compared to corresponding vehicle controls (IP-Veh vs. IP-STZ-3W, *p* > 0.05; IP-Veh vs. IP-STZ-6W, *p* < 0.01; ICV-Veh vs. ICV-STZ-3W, *p* < 0.01, and ICV-Veh vs. ICV-STZ-6W, *p* < 0.01) (Figure 2B). This could perhaps be due to STZ-induced toxicity, hyperglycemia/diabetes, or impaired insulin signaling in the hippocampus. In addition, a more significant deterioration in memory loss was observed in ICV-STZ-injected rats compared to the IP-STZ group (IP-STZ-3W vs. ICV-STZ-3W, *p* < 0.01; IP-STZ-6W vs. ICV-STZ-6W, *p* < 0.01) (Figure 2B).

### 3.3. IP-STZ and ICV-STZ Induced Early Changes in the Activity of AChE and Na^+^/K^+^-ATPase

**Figure 3:**
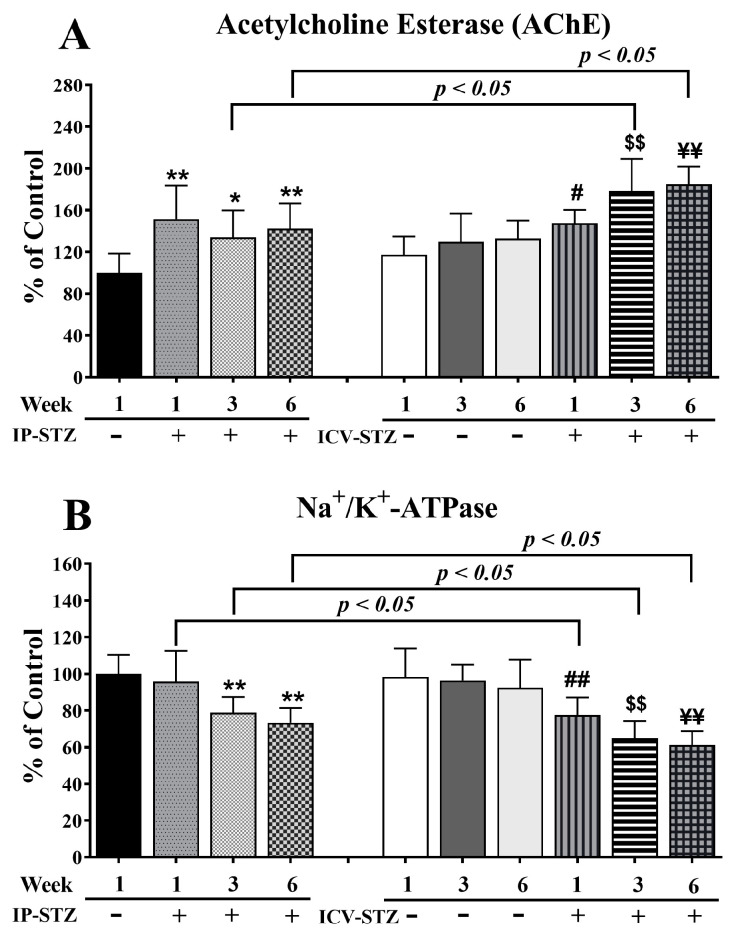
AChE (**A**) and Na^+^/K^+^-ATPase (**B**) activity in STZ- and vehicle-treated (IP or ICV) rats. There was a time-dependent increase in AChE and a significant decrease in Na^+^/K^+^-ATPase in both the IP-STZ and IVC-STZ groups compared to respective vehicle-treated control groups. IP-Veh vs. other groups, * *p* < 0.05, ** *p* < 0.01; ICV-Veh-1W vs. ICV-STZ-1W, # *p* < 0.05, ## *p* < 0.01; ICV-Veh-3W vs. ICV-STZ-3W, $$ *p* < 0.01; ICV-Veh-6W vs. ICV-STZ-6W, ¥¥ *p* < 0.01. These changes were more profound in ICV-STZ groups compared to respective IP-STZ groups (*p* < 0.05). Data are mean ± SD of *n* = 10 rats/group.

AChE breaks down ACh and maintains its optimal levels in the synapse. STZ administration (IP or ICV) significantly increased AChE activity (F (9, 90) = 13.23, *p* < 0001, Figure 3A) relative to the corresponding vehicle controls. IP-STZ treatment for 1W demonstrated the maximum increase in AChE activity compared to the IP-Veh (*p* < 0.01), IP-STZ-3W (*p* < 0.05), and IP-STZ-6W (*p* < 0.01) groups. STZ administration via the ICV route resulted in a profound increase in AChE activity relative to the IP route (F (3, 90) = 8.32; *p* < 0.0005)). The levels of AChE in ICV-STZ-treated animals were significantly higher at 3W (*p* < 0.05) and at 6W (*p* < 0.05) compared to IP-STZ-treated rats at 3W and 6W, respectively. Furthermore, the ICV administration of STZ significantly increased AChE activity in a time-dependent manner at 1W (30.2%, *p* < 0.05), 3W (48.5%, *p* < 0.01), and 6W (52.6%, *p* < 0.01) compared to ICV-vehicle-treated rats. There was no significant difference in AChE activity between the ICV-Veh and IP-Veh groups (*p* > 0.05).

Na^+^/K^+^-ATPase is an important enzyme that regulates the depolarization of cells/synapses, including the induction of LTP in the hippocampus. STZ administration, IP or ICV, significantly reduced the activity of Na^+^/K^+^-ATPase over time (F (9, 90) = 16.11, *p* < 0001, Figure 3B). In IP-STZ-treated rats, Na^+^/K^+^-ATPase activity was not significantly different from the vehicle control at 1W; however, it had significantly declined at 3W and 6W (*p* < 0.01). In contrast, in ICV-STZ-treated rats, Na^+^/K^+^-ATPase activity declined significantly starting at 1W (77.62%, *p* < 0.01), with an additional decrease observed at 3W (65%, *p* < 0.01) and 6W (61.23%, *p* < 0.01). At all three time points of 1W, 3W, and 6W, Na^+^/K^+^-ATPase activity in ICV-STZ-treated animals was significantly lower (*p* < 0.05) compared to the IP-STZ groups (F (3, 90) = 38.93; *p* < 0.0001). There was no significant change in Na^+^/K^+^-ATPase in any of the ICV-Veh- or IP-Veh-treated groups (*p* > 0.05). Further, there was also no significant difference when the ICV-Veh groups were compared with IP-Veh rats at any time point after injection (1W, 3W, and 6W).

### 3.4. IP-STZ and ICV-STZ Induced Early Changes in the Expression Levels and Activation States of PI3K/Akt/GSK-3β and PAK/LIMK-1/Cofilin-1 Signaling Pathways

**Figure 4:**
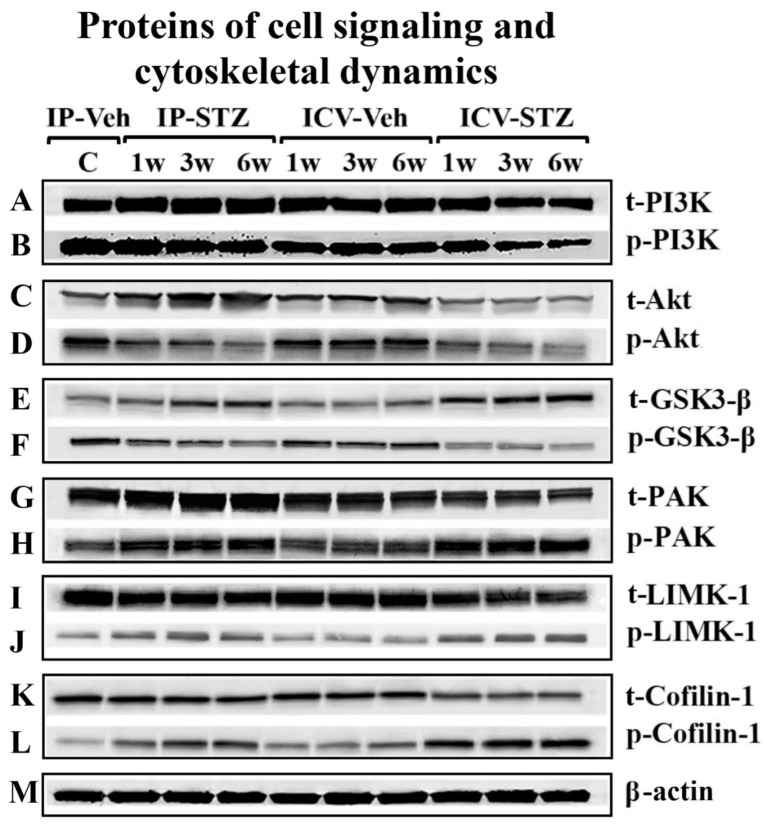
Representative immunoblot of mediators of neuronal survival and synaptic dynamics/plasticity, (**A**) t-PI3K, (**B**) p-PI3K, (**C**) t-Akt, (**D**) p-Akt, (**E**) t-GSK-3β, (**F**) GSK-3β, (**G**) t-PAK, (**H**) p-PAK, (**I**) t-LIMK-1, (**J**) p-LIMK-1, (**K**) t-cofilin-1, (**L**) p-cofilin-1, and (**M**) β-actin, as a loading control, in hippocampal synaptosomes.

To assess the early effects of impaired insulin signaling on the PI3K/Akt/GSK-3β and PAK/LIMK/cofilin pathways, the levels of phosphorylated (p) and total (t) protein isoforms were evaluated by immunoblotting in hippocampal synaptosomes from IP-STZ- and ICV-STZ-treated rats (Figure 4A–M). In the IP-STZ group, the levels of t-PI3K, t-Akt, and t-GSK-3β increased in a time-dependent manner. However, in the ICV-STZ group, t-PI3K and t-Akt decreased at 3W and 6W, while t-GSK-3β increased at 1W, 3W, and 6W. In the IP-STZ group, the level of total t-PAK significantly increased at 3W and 6W, while t-LIMK-1 and t-cofilin-1 decreased in a time-dependent manner. The levels of phosphorylated isoforms, p-PI3K, p-Akt, and p-GSK-3β and the ratio of phospho- to total protein (p-PI3K/t-PI3K, p-Akt/t-Akt, and p-GSK-3β/t-GSK-3β) time-dependently decreased in STZ-treated rats. In contrast, the levels of p-PAK, p-LIMK-1, and p-cofilin-1 and their ratio to total isoforms (p-PAK/t-PAK, p-LIMK-1/t-LIMK-1, and p-cofilin-1/t-cofilin-1) increased in a time-dependent manner in response to STZ treatment.

**Figure 5:**
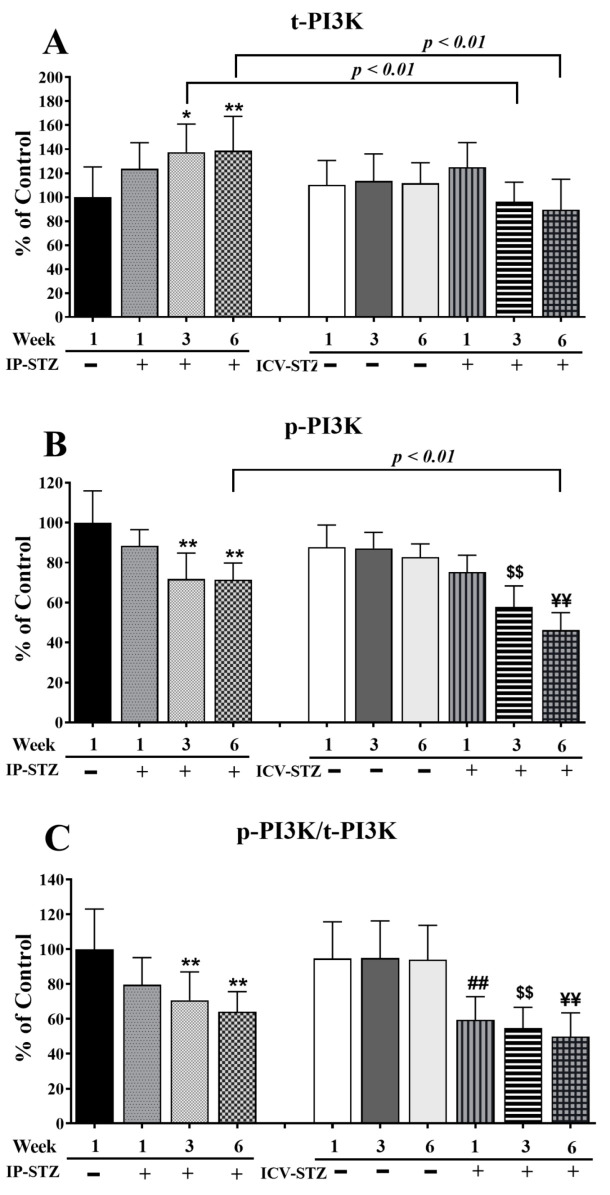
Quantification of t-PI3K (**A**), p-PI3K (**B**), and p-PI3K/t-PI3K (**C**) levels in hippocampal synaptosomal fractions from different groups. The t-PI3K, p-PI3K, and p-PI3K/t-PI3K levels increased in a time-dependent manner in both IP-STZ and IVC-STZ groups compared to respective vehicle control groups. t-PI3K was significantly higher in IP-STZ groups compared to ICV-STZ groups. p-PI3K and p-PI3K/t-PI3K were, on the other hand, significantly lower in the IP-STZ groups compared to the ICV-STZ groups. IP-Veh vs. other groups, * *p* < 0.05, ** *p* < 0.01; ICV-Veh vs. ICV-STZ-1W, ## *p* < 0.01; ICV-Veh-3W vs. ICV-STZ-3W, $$ *p* < 0.01; ICV-Veh-6W vs. ICV-STZ-6W, ¥¥ *p* < 0.01. Data are mean ± SD of *n* = 10 rats/group.

The level of t-PI3K markedly increased (F (9, 90) = 65.86; *p* < 0.0001) in the IP-STZ group (Figure 5A) but remained unchanged in the ICV-STZ group. The levels of p-PI3K, however, were significantly reduced (F (9, 90) = 100.3; *p* < 0.0001) (Figure 5B). The ratio of p-PI3K to t-PI3K decreased in both STZ-treated groups as compared to the vehicle controls (F (9, 90) = 34.07; *p* < 0.0001) (Figure 5C). A small increase in t-PI3K was observed one-week post-STZ injection IP (IP-STZ-1W; 123.61% of control (*p* > 0.05)), which reached a maximal level by 6W (IP-STZ-6W; 138% of control (p < 0.01)). t-PI3K levels decreased in the ICV-STZ group at 6W; however, this decrease was not significantly different compared to the ICV vehicle group (ICV-STZ-6W; 89% (*p* > 0.05)). Moreover, t-PI3K levels at 1W were not significantly different between the IP-STZ and ICV-STZ groups. Nonetheless, t-PI3K protein levels in the ICV-STZ group at 3W and 6W were significantly (*p* < 0.01) lower compared to the corresponding time points in the IP-STZ group (F (3, 90) = 14.39; *p* < 0.0001). p-PI3K levels declined within one-week post-STZ injection (IP-STZ-1W; 88.4% of control (*p* > 0.07)) and reached a minimal level by 6W in the IP-STZ (IP-STZ-6W; 71.85% (*p* < 0.05)) and ICV-STZ (ICV-STZ-6W; 71.24% (*p* < 0.01)) groups. There was no significant difference in p-PI3K between the IP-STZ and ICV-STZ groups at 1W and 3W. However, p-PI3K levels in ICV-STZ-treated animals were significantly lower at 6W (*p* < 0.01) compared to the corresponding time point in the IP-STZ group (F (3, 90) = 62.8; *p* < 0.0001). There was no significant difference in p-PI3K levels between vehicle groups (IP or ICV) at 1W and 3W; however, significant differences were observed at 6W. The main treatment effect of STZ was time-dependent in p-PI3K/t-PI3K (F (3, 90) = 46.93; *p* < 0.0001); however, none of the groups showed significant differences between the IP and ICV routes of STZ administration.

**Figure 6:**
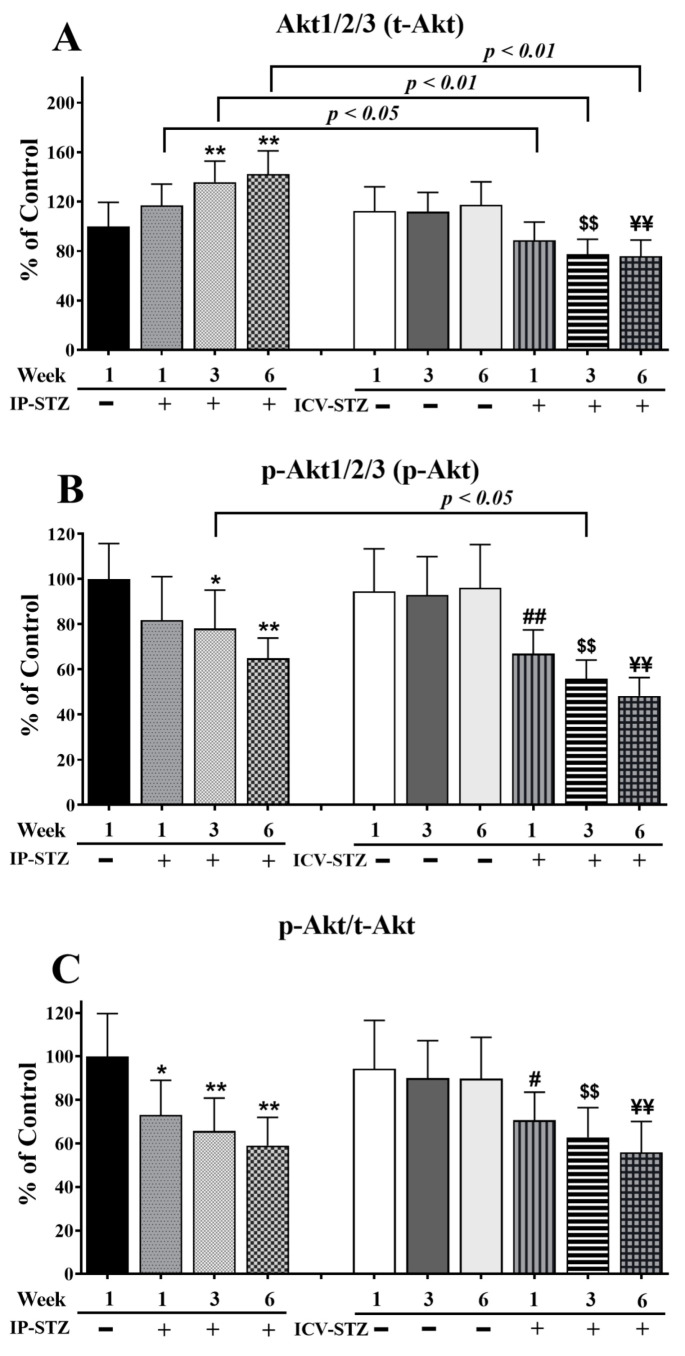
Quantification of t-Akt (**A**), p-Akt (**B**), and p-Akt/t-Akt (**C**) levels in hippocampal synaptosomal fractions from different groups. The t-Akt, p-Akt, and p-Akt/t-Akt expression was altered in a time-dependent manner in both IP-STZ and ICV-STZ groups. t-Akt was significantly higher in IP-STZ groups compared to ICV-STZ groups. p-Akt and p-Akt/t-Akt were, on the other hand, significantly lower in the IP-STZ groups compared to the ICV-STZ groups. IP-Veh vs. other groups, * *p* < 0.05, ** *p* < 0.01; ICV-Veh vs. ICV-STZ-1W, # *p* < 0.05, ## *p* < 0.01; ICV-Veh-3W vs. ICV-STZ-3W, $$ *p* < 0.01; ICV-Veh-6W vs. ICV-STZ-6W, ¥¥ *p* < 0.01. Data are mean ± SD of *n* = 10 rats/group.

Similar to t-PI3K, t-Akt significantly increased (F (9, 90) = 65.37; *p* < 0.0001) in the IP-STZ group (Figure 6A) and decreased in the ICV-STZ group. The levels of p-Akt (F (9, 90) = 61.85; *p* < 0.0001) and the ratio of p-Akt/t-Akt (F (9, 90) = 36.88; *p* < 0.0001) decreased in all STZ-treated groups compared to vehicle controls (Figure 6B,C). The main treatment effect of STZ on the t-Akt levels was time-dependent. In the IP-STZ group, a clear increase in t-Akt was observed within 1W (IP-STZ-1W; 117 % of control), which reached a maximal level by 6W (IP-STZ-6W; 142.2% of control (*p* < 0.01)). In the ICV-STZ group, however, t-Akt significantly decreased over time post-injection: 1W (*p* < 0.05), 3W (*p* < 0.01), and 6W (*p* < 0.01) compared to the corresponding time points in the IP-STZ group (F (3, 90) = 49.26; *p* < 0.001). There was no significant difference in t-Akt levels between vehicle-treated groups (IP-Veh and ICV-Veh).

p-Akt decreased one-week post-STZ injection (IP-STZ-1W; 81.85% (*p* > 0.05)) and reached a minimal level by 6W in both the IP-STZ (IP-STZ-6W; 64.92% (*p* < 0.01)) and ICV-STZ (ICV-STZ-6W; 48.24% (*p* < 0.01)) groups. There was no significant difference in the levels of p-Akt between the IP and ICV injection of STZ at 1W and 6W; however, the p-Akt levels in ICV-STZ-injected rats were significantly lower at 3W (*p* < 0.01) compared to IP-STZ-3W (F (3, 90) = 49.58; *p* < 0.001). Vehicle-treated groups did not show significant differences over time or between routes of STZ administration. Furthermore, the ratio of phosphorylated to total Akt (p-Akt/t-Akt) was comparable to the result for p-Akt (F (3, 90) = 37.30; *p* < 0.001); nevertheless, it was not affected by time or the route of injection

**Figure 7:**
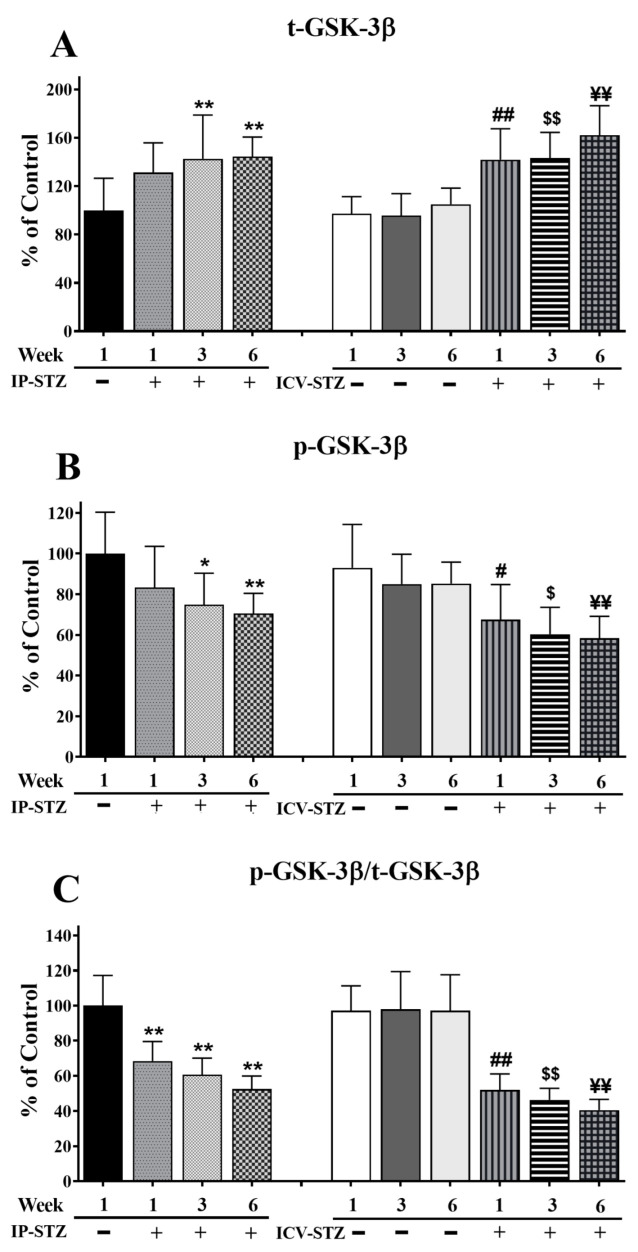
Quantification of t-GSK-3β (**A**), p-GSK-3β (**B**), and p-GSK-3β/t-GSK-3β (**C**) levels in hippocampal synaptosomal fractions. The t-GSK-3β, p-GSK-3β, and p-GSK-3β/t-GSK-3β levels were altered in a time-dependent manner in both IP-STZ and IVC-STZ groups compared to respective vehicle controls. t-GSK-3β significantly increased in IP-STZ and IVC-STZ groups compared to respective vehicle controls. p-GSK-3β and p-GSK-3β/t-GSK-3β, however, significantly decreased in IP-STZ and IVC-STZ groups. ICV administration of STZ induced more profound changes relative to the IP route. IP-Veh vs. other groups, * *p* < 0.05, ** *p* < 0.01; ICV-Veh vs. ICV-STZ-1W, # *p* < 0.05, ## *p* < 0.01; ICV-Veh-3W vs. ICV-STZ-3W, $ *p* < 0.05, $$ *p* < 0.01; ICV-Veh-6W vs. ICV-STZ-6W, ¥¥ *p* < 0.01. Data are mean ± SD of *n* = 10 rats/group.

The levels of t-GSK-3β significantly increased in a time-dependent manner in both IP and ICV STZ-treated animals (F (9, 90) = 91.57, *p* < 0001) (Figure 7A). The levels of p-GSK-3β (F (9, 90) = 57.08; *p* < 0.0001) (Figure 7B) and the ratio of p-GSK-3β/t-GSK-3β (F (9, 90) = 61.90; *p* < 0.0001) (Figure 7C) decreased time-dependently in all STZ-treated groups as compared to respective vehicle controls. No significant changes in t-GSK-3β levels were observed one week after the IP injection of STZ (21.5%; *p* > 0.05); nonetheless, t-GSK-3β levels declined significantly by 3W and 6W (*p* < 0.01). STZ administration via the ICV route was more potent in increasing t-GSK-3β levels compared to the IP route (F (3, 90) = 40.99; *p* < 0.001). The levels of t-GSK-3β in the ICV-STZ group were significantly higher (*p* < 0.01) at all three time points compared to the respective ICV-Veh group. However, there was no significant difference in t-GSK-3β levels between ICV- and IP-treated (vehicle or STZ) groups (*p* > 0.05), at any time point (1W, 3W and 6W) post-injection. p-GSK-3β decreased within one-week post-STZ injection (IP-STZ-1W; 83.37% (*p* > 0.05)) and continued to decrease until 6W, in both the IP-STZ (IP-STZ-6W; 70.54% (*p* < 0.01)) and ICV-STZ (ICV-STZ-6W; 58.34% (*p* < 0.01)) groups. The treatment method (IP versus ICV) did not show any significant effects at any time, 1W, 3W, or 6W, post-STZ injection. Comparatively, a significant decline in t-GSK-3β was observed when STZ was injected ICV (F (3, 90) = 30.53; *p* < 0.001). The changes in p-GSK-3β/t-GSK-3β were time-dependent and comparable to the results for p-GSK-3β (F (3, 90) = 108.08; *p* < 0.001). The levels of p-GSK-3β or p-GSK-3β/t-GSK-3β were not significantly different between the IP and ICV groups (*p* > 0.05).

**Figure 8:**
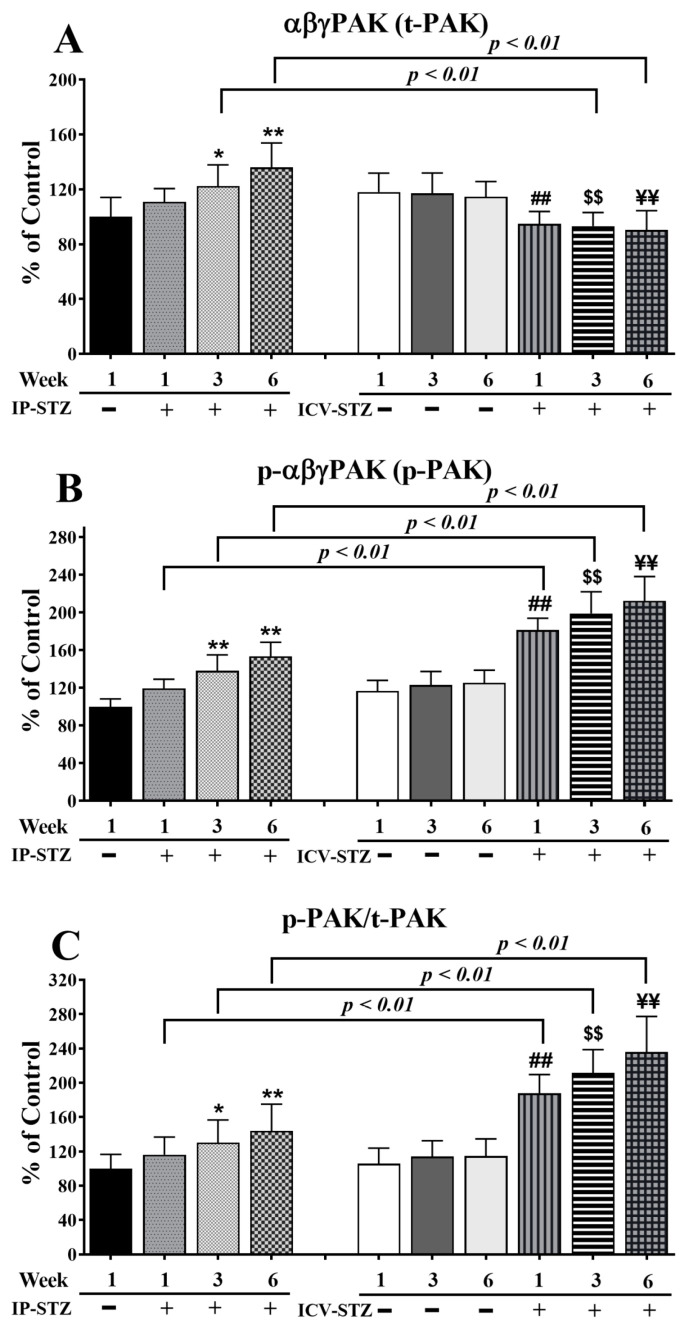
Changes in t-PAK, p-PAK, and p-PAK/t-PAK levels analyzed in hippocampal synaptosomal fractions. Time-dependent significant changes in t-PAK (**A**), p-PAK (**B**), and p-PAK/t-PAK (**C**) in IP-STZ and IVC-STZ groups. t-PAK was significantly higher in IP-STZ groups compared to ICV-STZ groups (*p* < 0.01). p-PAK and p-PAK/t-PAK were, on the other hand, significantly lower in the IP-STZ groups compared to the ICV-STZ groups (*p* < 0.01). IP-Veh vs. other groups, * *p* < 0.05, ** *p* < 0.01; ICV-Veh vs. ICV-STZ-1W, ## *p* < 0.01; ICV-Veh-3W vs. ICV-STZ-3W, $$ *p* < 0.01; ICV-Veh-6W vs. ICV-STZ-6W, ¥¥ *p* < 0.01. Data are mean ± SD of *n* = 10 rats/group.

Quantitative analysis of the blots showed that the levels of t-PAK were relatively unaffected by vehicle injections (IP or ICV) at all time points (*p* > 0.05). Nevertheless, IP-STZ significantly increased t-PAK expression in hippocampal synaptosomes at 3W (*p* < 0.05) and 6W (*p* < 0.01) (F (9, 90) = 80.58, *p* < 0001) (Figure 8A). ICV-STZ injection, however, significantly (*p* < 0.01) reduced t-PAK levels in all STZ-treated groups (1W, 3W, and 6W) compared to the respective ICV-Veh groups, and these values were not different from those of the IP-Veh control group. This could have been due to the increased basal levels of t-PAK in the ICV-Veh groups. A marked effect of the treatment route was observed on t-PAK expression (F (3, 90) = 30.78; *p* < 0.001). The t-PAK levels in the IP-STZ groups were significantly different from the ICV-STZ group, at 3W and 6W (*p* < 0.01). The levels of p-PAK (F (9, 90) = 141.7; *p* < 0.0001) (Figure 8B) and the ratio of p-PAK/PAK (F (9, 90) = 73.25; *p* < 0.0001) (Figure 8C) increased in a time-dependent manner in all STZ-treated groups compared to their respective controls. The levels of p-PAK started to increase one-week post-STZ injection (IP-STZ-1W; 119.4% of control (*p* > 0.05)) and reached a maximal level by 6W, with both treatment methods: IP-STZ (IP-STZ-6W; 153.6% of control (*p* < 0.01)) and ICV-STZ (ICV-STZ-6W; 212.3% of control (*p* < 0.01)). The ICV-Veh group, at 6W, showed a significant increase (*p* < 0.05) in p-PAK (as p-PI3K decreased only in this group). In all other vehicle groups, the levels of p-PAK and their ratios to t-PAK (p-PAK/t-PAK) were unaffected. A significantly exacerbated treatment effect on p-PAK (F (3, 90) = 182.2; *p* < 0.001) and p-PAK/t-PAK (F (3, 90) = 131.4; *p* < 0.001) was observed when STZ was injected ICV. Significant (*p* < 0.01) differences in p-PAK and p-PAK/t-PAK were observed between the ICV-STZ and IP-STZ groups. There was no significant difference between the IP vehicle and ICV vehicle at any time post-injection (1W, 3W, or 6W).

**Figure 9:**
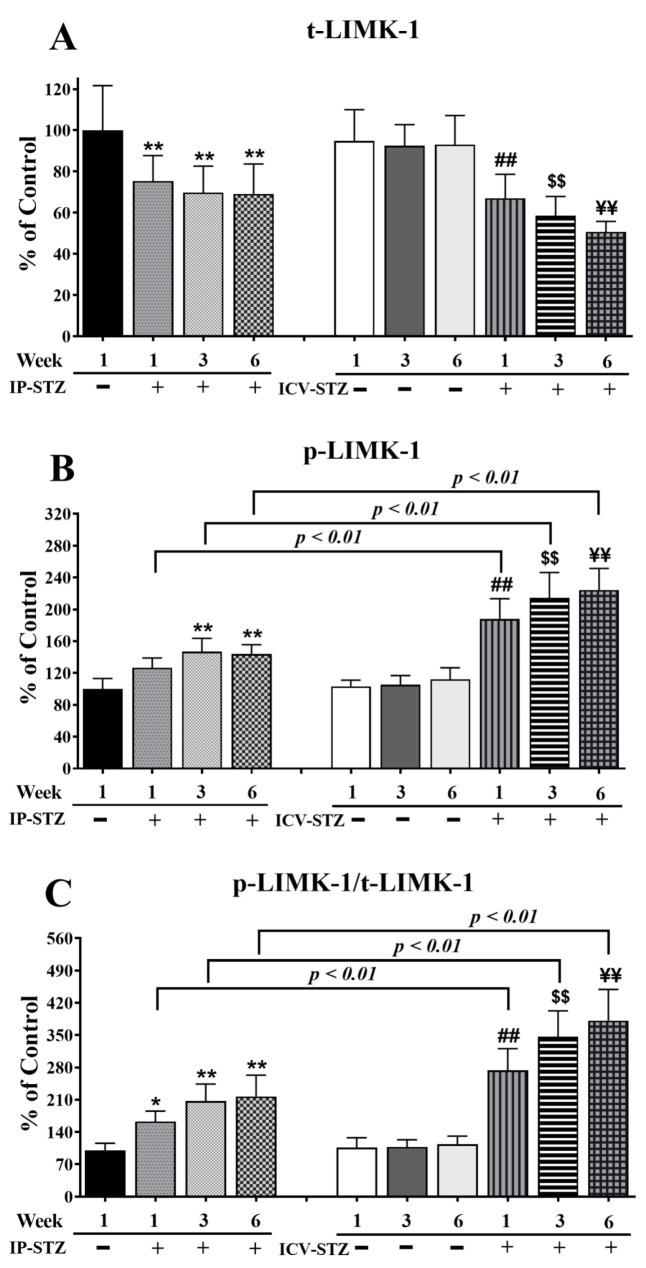
Time course of changes in t-LIMK-1 (**A**), p-LIMK-1 (**B**), and p-LIMK-1/t-LIMK-1 (**C**) in hippocampal synaptosomes. Time-dependent significant changes in t-LIMK-1, p-LIMK-1, and p-LIMK-1/t-LIMK-1 in IP-STZ and IVC-STZ groups as compared to respective vehicle controls. t-LIMK-1 significantly decreased, while p-LIMK-1 and p-LIMK-1/t-LIMK-1 significantly increased in IP-STZ and ICV-STZ groups. The ICV route was more potent than the IP route in inducing these changes. IP-Veh vs. other groups, * *p* < 0.05, ** *p* < 0.01; ICV-Veh vs. ICV-STZ-1W, ## *p* < 0.01; ICV-Veh-3W vs. ICV-STZ-3W, $$ *p* < 0.01; ICV-Veh-6W vs. ICV-STZ-6W, ¥¥ *p* < 0.01. Data are mean ± SD of *n* = 10 rats/group.

In addition, time course analysis revealed that the levels of total (t-LIMK-1) and phosphorylated LIMK-1 (p-LIMK-1) were unaffected by the vehicle at any time point after IP or ICV injection (*p* > 0.05). The levels of t-LIMK-1 decreased in a time-dependent manner in the IP-STZ group (IP-STZ-1W; 75.3% (*p* < 0.01), IP-STZ-3W; 70.75% (*p* < 0.01), and IP-STZ-6W; 68% (*p* < 0.01)) and in the ICV-STZ group (ICV-STZ-1W; 67% (*p* < 0.01), ICV-STZ-1W; 58.53% (*p* < 0.01), and ICV-STZ-1W; 50.64% (*p* < 0.01)) (F (9, 90) = 87.70, *p* < 0001) (Figure 9A). The levels of p-LIMK-1 (F (9, 90) = 130.6; *p* < 0.0001) (Figure 9B) and the ratio of p-LIMK-1/t-LIMK-1 (F (9, 90) = 140.3; *p* < 0.0001) (Figure 9C) increased in a time-dependent manner in STZ-treated rats compared to respective controls. Similar to p-PAK, p-LIMK-1 levels increased one-week post-STZ injection (IP-STZ-1W; 126% (*p* > 0.05)) and reached a maximal level by 6W in the IP (IP-STZ-6W; 143.9% (p < 0.01)) and ICV (ICV-STZ-6W; 224% (*p* < 0.01)) groups. The administration of STZ via the ICV route significantly increased p-LIMK-1 (F (3, 90) = 190.1; *p* < 0.0001) and p-LIMK-1/t-LIMK-1 (F (3, 90) = 268.5; *p* < 0.0001) as compared to the IP route. The levels of p-LIMK-1 and the ratio of p-LIMK-1/t-LIMK-1 were significantly (*p* < 0.01) different between the ICV-STZ and IP-STZ groups. LIMK-1 levels, however, were not different between the IP and ICV vehicle groups at any time (1W, 3W, or 6W) post-injection.

**Figure 10:**
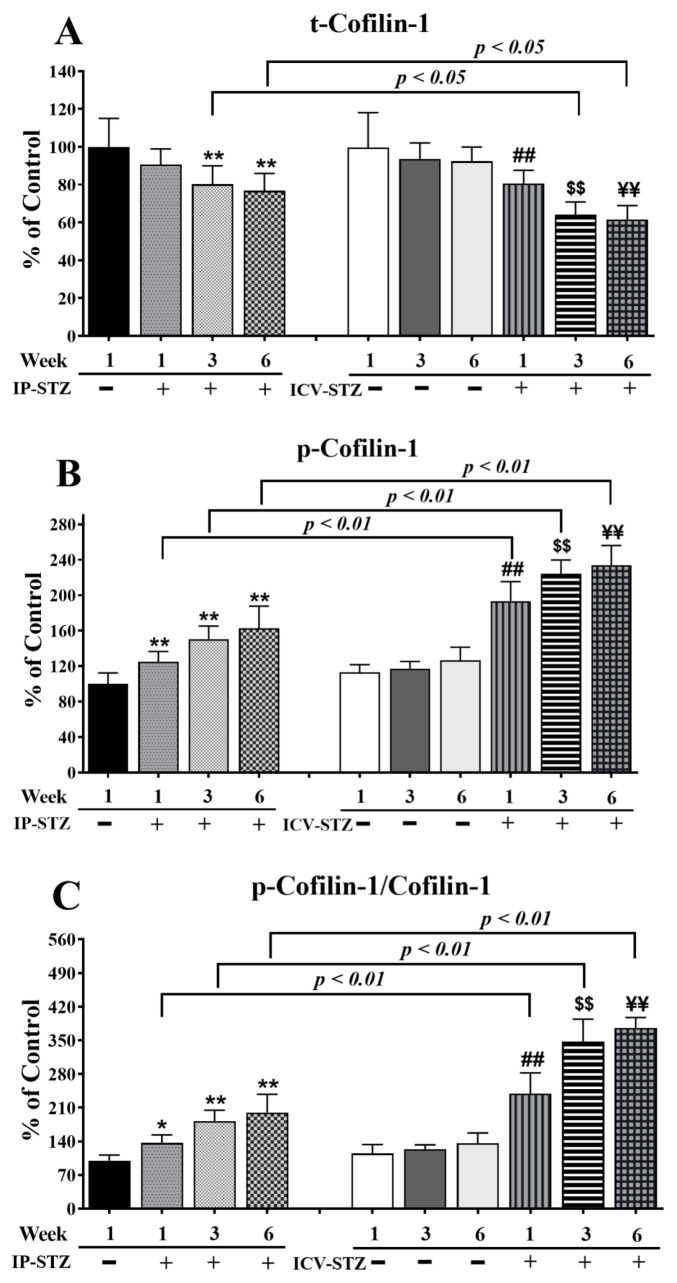
Changes in the levels of t-cofilin-1, p-cofilin-1, and p-cofilin-1/t-cofilin-1 in response to IP and ICV administration of STZ in hippocampal synaptosomes. Time course analysis showed significant alterations in the levels of t-cofilin-1 (**A**), p-cofilin-1 (**B**), and p-cofilin-1/t-cofilin-1 (**C**) in IP-STZ and IVC-STZ groups compared to corresponding vehicle controls. t-cofilin-1 levels significantly decreased, while p-cofilin-1 and p-cofilin-1/t-cofilin-1 significantly increased in IP-STZ and ICV-STZ groups (*p* < 0.01). The administration of STZ through the ICV route was more potent than the IP route in inducing these changes. IP-Veh vs. other groups, * *p* < 0.05, ** *p* < 0.01; ICV-Veh vs. ICV-STZ-1W, ## *p* < 0.01; ICV-Veh-3W vs. ICV-STZ-3W, $$ *p* < 0.01; ICV-Veh-6W vs. ICV-STZ-6W, ¥¥ *p* < 0.01. Data are mean ± SD of *n* = 10 rats/group.

Finally, time course analysis revealed that changes in cofilin-1 expression mirrored the alterations observed in LIMK-1. The levels of total cofilin-1 (t-cofilin-1) and phosphorylated cofilin-1 (p-cofilin-1) were not significantly affected in vehicle-treated rats following IP or ICV injection at all time points tested. The levels of t-cofilin-1 significantly and time dependently decreased in the IP-STZ and ICV-STZ groups (F (9, 90) = 86.93, *p* < 0001) (Figure 10A). The main treatment effect revealed that ICV-STZ injection exerted a severe impact on t-conflin-1 (F (3, 90) = 41.06; *p* < 0.0001), p-cofilin-1 (F (3, 90) = 256.7; *p* < 0.0001), and p-cofilim-1/t-cofilin-1 (F (3, 90) = 390.6; *p* < 0.0001) levels compared to IP-STZ injection. Moreover, time course analysis revealed that the levels of p-cofilin-1 (F (9, 90) = 159.2; *p* < 0.0001) (Figure 10B) and p-cofilin-1/t-cofilin-1 (F (9, 90) = 176.7; *p* < 0.0001) (Figure 10C) were significantly augmented in all STZ-treated groups compared to vehicle controls. p-cofilin-1 expression was upregulated one-week post-STZ injection (IP-STZ-1W; 124.9% of control (*p* > 0.01)) and reached a maximal level by 6W in both the IP (IP-STZ-6W; 162.7% of control (*p* < 0.01)) and ICV groups (ICV-STZ-6W; 234% of control (*p* < 0.01)). The levels of p-cofilin-1 and the ratio of p-cofilin-1/t-cofilin-1 were significantly different between the ICV-STZ and IP-STZ groups (*p* < 0.01) at all times post-injection.

### 3.5. IP-STZ and ICV-STZ Induced Changes in Mediators of Neuronal Survival and Synaptic Dynamics/Plasticity Correlated with Cognitive Impairments

**Figure 11:**
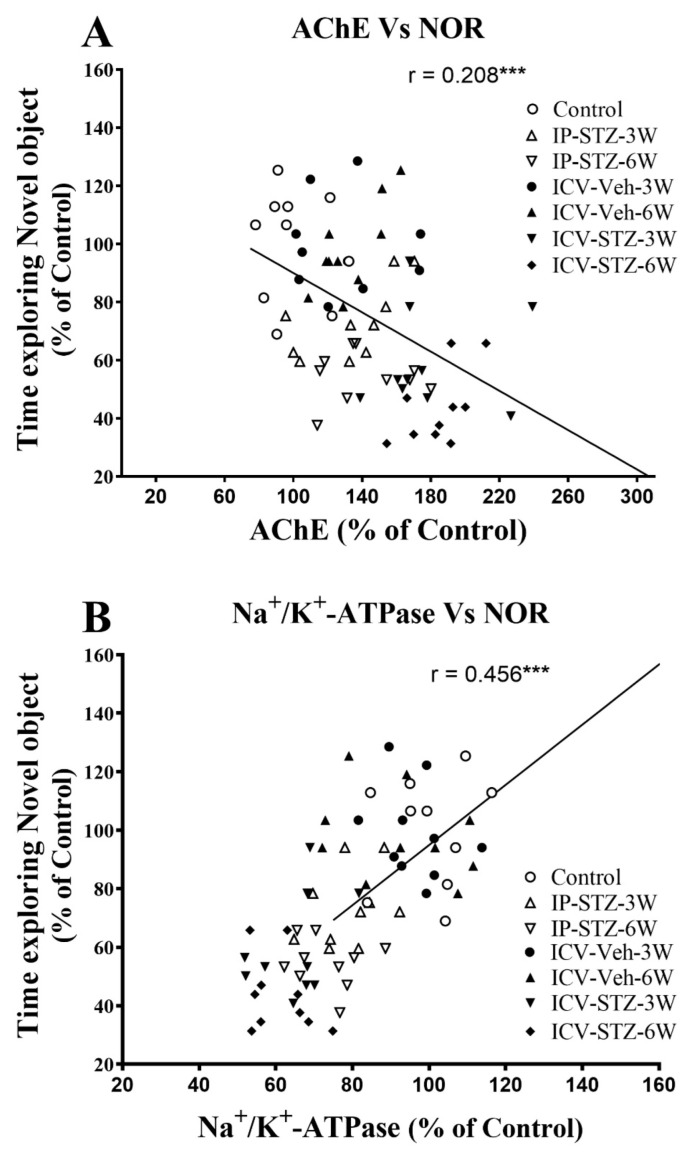
Correlation between AChE (**A**) and Na^+^/K^+^-ATPase (**B**) activity and cognitive performance (time exploring novel object during T2 test in NOR) in response to STZ administration. Increased acetylcholine metabolizing enzyme (AChE) activity correlated negatively with decreased time spent exploring novel object (r = 0.208), tested in T2 session. Decreased Na^+^/K^+^-ATPase (membrane/synapse potentiation marker) activity correlated positively with the procurement of memory to explore the novel object (r = 0.456). *** *p* < 0.0001, *n* = 10 rats/group at 3W and 6W.

**Figure 12:**
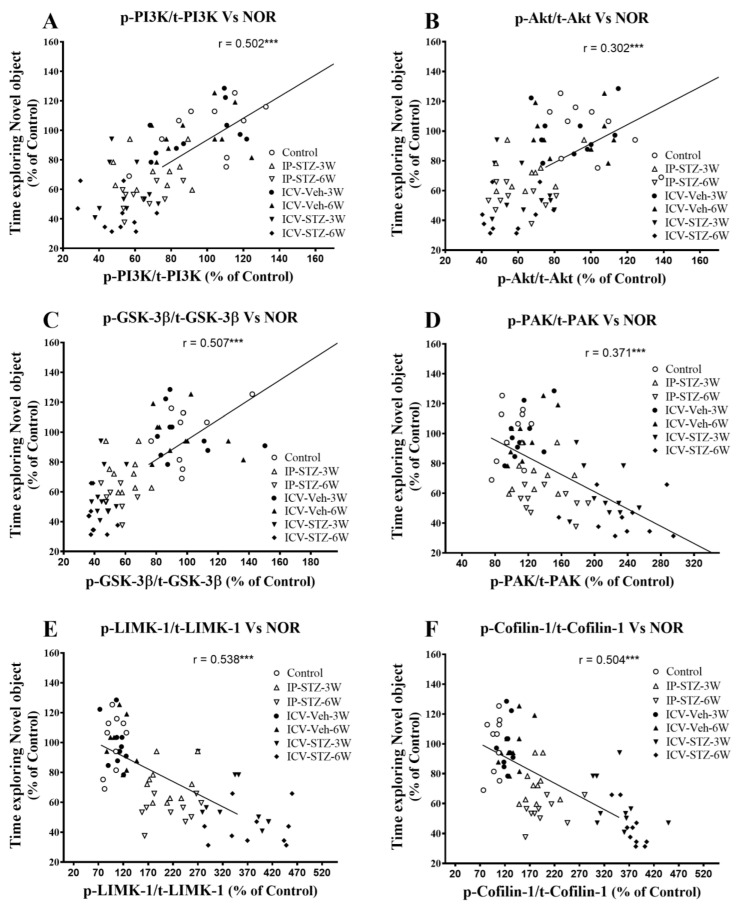
Correlation between PI3K and PAK signaling mediators (declined p-PI3K/t-PI3K (**A**), p-Akt/t-Akt (**B**), p-GSK-3β/t-GSK-3β (**C**), and increased p-PAK/t-PAK (**D**), p-LIMK-1/t-LIMK-1 (**E**) and p-cofilin-1/t-cofilin-1 (F)) and time exploring novel object in NOR test. Decreased levels of p-PI3K/t-PI3K (r = 0.502), p-Akt/t-Akt (r = 302), and p-GSK-3β/t-GSK-3β (r = 507) correlated positively with decreased time spent exploring novel object (**A**–**C**), tested in T2 (with N-object) after the last session in T1 (with F-object) on the 4th day. Increased levels of p-PAK/t-PAK (r = 0.371), p-LIMK-1/t-LIMK-1 (r = 0.538), and p-cofilin-1/t-cofilin-1 (r = 0.504) negatively correlated with a decline in time exploring novel object (**D**–**F**). *** *p* < 0.0001, *n* = 10 rats/group at 3W and 6W.

We evaluated the possible relationship between changes in the activity of AChE and Na^+^/K^+^-ATPase and the PI3K and PAK signaling pathways and rats’ cognitive performance (time exploring novel object (NOR task) and incorrect response in T-maze (as working memory impairment)). As the activity of AChE increased in hippocampal synaptosomes, the time spent exploring novel objects significantly decreased (r = 0.208; *p* < 0.001, Figure 11A). Moreover, as the activity of Na^+^/K^+^-ATPase decreased, the time spent exploring novel objects significantly decreased (r = 0.456; *p* < 0.001, Figure 11B). In addition, we evaluated the association between cognitive performance and the levels of total and phosphorylated hippocampal synaptosomal elements of the PI3K and PAK pathways. The association of different variables was similar when analyzed separately. Data presented here refer to the association between the cognitive status and changes in the ratios of phosphorylated to total protein. There was a significant association between impairments in exploration behavior and a decreased ratio of p-PI3K/t-PI3K (r = 0.502; Figure 12A), p-Akt/t-Akt (r = 0.302; Figure 12B), and p-GSK-3β/t-GSK-3β (r = 0.509; Figure 12C) and an increased ratio of p-PAK/t-PAK (r = 0.371; Figure 12D), p-LIMK-1/t-LIMK-1 (r = 0.538; Figure 12E), and p-cofilin-1/t-cofilin-1 (r = 0.504; Figure 12F) in hippocampal synaptosomes following STZ injection.

**Figure 13:**
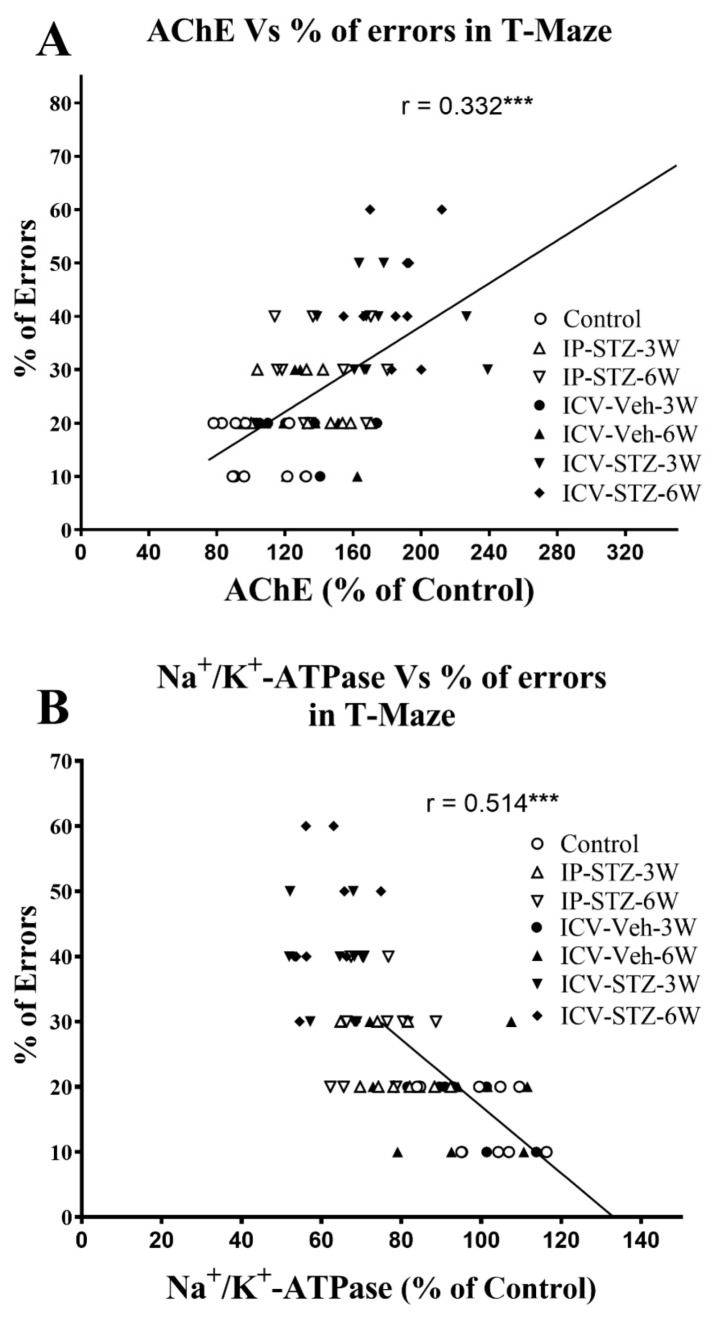
Correlation between AChE (**A**) and Na^+^/K^+^-ATPase (**B**) activity and cognitive performance (working memory during probe test in T-maze) in response to STZ administration. Increased AChE activity correlated negatively with decreased correct choices and increased percentage of errors (r = 0.332), tested 1 h after the last learning session. Decreased Na^+^/K^+^-ATPase (membrane/synapse potentiation marker) activity correlated positively with working memory. As Na^+^/K^+^-ATPase activity decreased, the percentage of errors in choosing a correct spontaneous alternate turn in the T-maze increased (r = 0.514). *** *p* < 0.0001, *n* = 10 rats/group at 3W and 6W.

**Figure 14:**
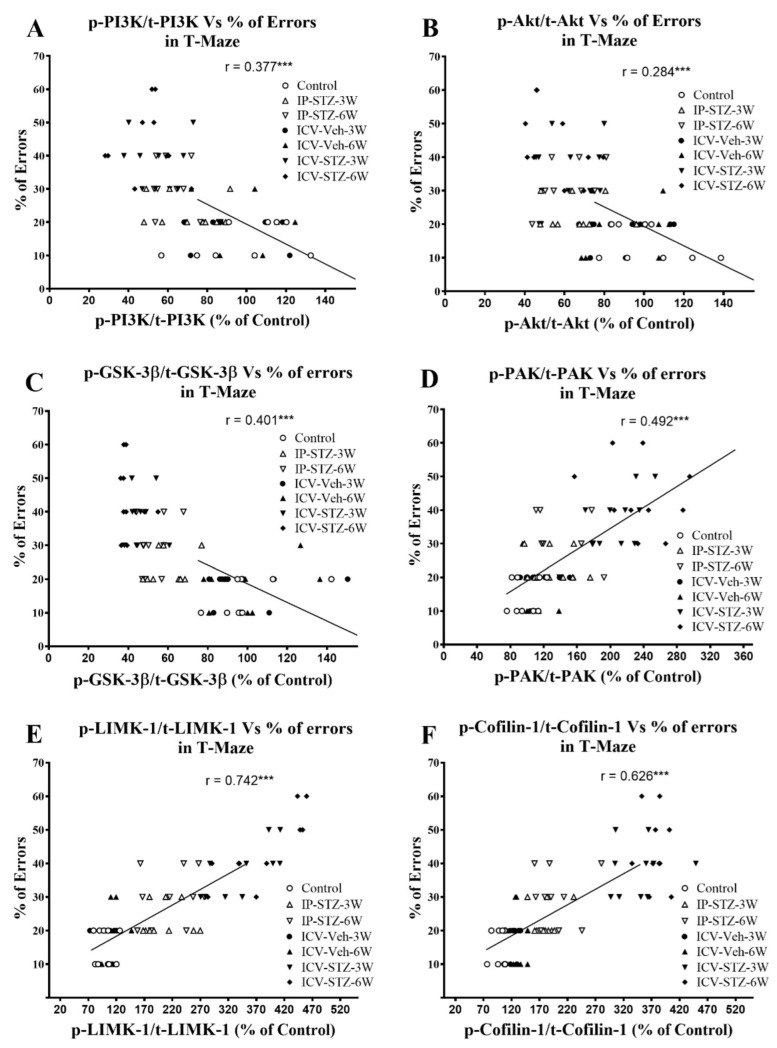
Correlation of changes in PI3K and PAK signaling mediators (declined p-PI3K/t-PI3K (**A**), p-Akt/t-Akt (**B**), p-GSK-3β/t-GSK-3β (**C**), and increased p-PAK/t-PAK (**D**), p-LIMK-1/t-LIMK-1 (**E**) and p-cofilin-1/t-cofilin-1 (**F**)) with percentage of errors in T-maze. Decreased levels of p-PI3K/t-PI3K (r = 0.377), p-Akt/t-Akt (r = 0.284), and p-GSK-3β/t-GSK-3β (r = 401) correlated negatively with decreased working memory procurement, tested 1 h after the last learning session (**A**–**C**). Increased levels of p-PAK/t-PAK (r = 0.492), p-LIMK-1/t-LIMK-1 (r = 0.742), and p-cofilin-1/t-cofilin-1 (r = 0.626) positively correlated with a decline in working memory (**D**–**F**), tested in T-maze. *** *p* < 0.0001, *n* = 10 rats/group at 3W and 6W.

Similarly, as the activity of AChE increased in hippocampal synaptosomes, cognitive impairments (% of errors) in the T-maze significantly increased (r = 0.332; *p* < 0.001, Figure 13A). Moreover, as the activity of Na^+^/K^+^-ATPase decreased, the % of errors in the T-maze significantly increased (r = 0.514; *p* < 0.001, Figure 13B). In addition, we evaluated the association between working memory and the levels of total and phosphorylated hippocampal synaptosomal elements of the PI3K and PAK pathways. There was a significant association between impairments in working memory and a decreased ratio of p-PI3K/t-PI3K (r = 0.377; Figure 14A), p-Akt/t-Akt (r = 0.284; Figure 14B), and p-GSK-3β/t-GSK-3β (r = 0.401; Figure 14C) and an increased ratio of p-PAK/t-PAK (r = 0.492; Figure 14D), p-LIMK-1/t-LIMK-1 (r = 0.742; Figure 14E), and p-cofilin-1/t-cofilin-1 (r = 0.626; Figure 14F) in hippocampal synaptosomes following STZ injection. The association of different variables with the percentage of errors in the T-maze was similar when analyzed separately.

## 4. Discussion

This study evaluated the effect of early impairments in insulin signaling on hippocampal synaptic dynamics/plasticity in IP-STZ- and ICV-STZ-treated rats. Time course assessments were performed at 1W, 3W, and 6W after STZ administration. IP and ICV STZ administration impaired rats’ cognition, as indicated by a lack of interest in recognizing novel objects and attenuated working memory. In addition, STZ treatment increased the activity of AChE, decreased the activity of Na^+^/K^+^-ATPase, and impaired the signaling pathways involved in neuronal survival and synaptic dynamics/plasticity, including the PI3K/Akt/GSK3β and PAK/LIMK-1/cofilin-1 pathways. As per our knowledge, this is the first study that has evaluated the early effects of impaired insulin signaling on markers of neurotransmission and synaptic potentiation (AChE and Na^+^/K^+^-ATPase activity) and hippocampal proteins regulating neuronal survival and synaptic dynamics/plasticity in the early course of the disease.

The NOR and T-maze tests are widely accepted techniques for the evaluation of cognitive behavior, as a form of non-spatial learning/memory [74,83]. The time spent exploring objects in the second trial of the fourth day decreased in STZ-treated rats, suggesting cognitive deficits expressed as a loss of interest and recognition (Figure 1). The percentage of incorrect choices decreased in vehicle-treated rats, suggesting improved learning, improved detection of correct choices, and enhanced working memory. This, however, was compromised upon STZ administration, in both the IP and ICV routes. Additionally, the ICV route exhibited profound, time-dependent, detrimental effects over the IP route (Figure 1B,C). Similarly, in the T-maze, STZ-treated rats demonstrated a significant and time-dependent deterioration in learning and memory, with a more profound effect observed in the ICV-STZ-treated rats (Figure 2). Our results support previous studies that showed that IP- or ICV-STZ-injected animals exhibited reduced cognitive ability [75,84,85,86] and support the deleterious effect of the ICV route of STZ administration [3]. Our data are consistent with previous studies reporting deteriorated cognition following impaired insulin signaling in rodents four weeks post-IP-STZ [87,88] and two weeks post-ICV-STZ administration [30]. Hyperglycemia results in encephalopathy and exacerbates other neurological conditions, including stroke [89] and AD. Insulin resistance and AD aggravate each other [90]. Chronic hyperglycemia not only aggravates changes in synaptic morphology and cognitive loss [91], but also may impair cytoprotective mechanisms [92].

AChE breaks down ACh to adjust its optimal levels in cholinergic synapses. Na^+^/K^+^-ATPase regulates cell membranes and synapse potentiation. Both enzymes are key regulators of cholinergic neurotransmission, nerve conduction, and cognitive function [24,93]. In the course of the disease, impaired insulin signaling in the synaptosomes is aggravated due to increased byproducts of lipid peroxidation [22], specifically in the hippocampus [3]. Hyperglycemia has been shown to have a detrimental effect on the binding of α-amino-3-hydroxy-5-methyl-4-isoxazolepropionic acid (AMPA) to glutamate receptor-1 (GluR1) in the hippocampus [23]. In addition, hyperglycemia reduces the expression of LTP in the hippocampus [26], attenuates Na^+^/K^+^-ATPase activity [24], and disrupts Ca^2+^-dependent synaptic potentiation [25]. The loss of LTP in the hippocampus results in cognitive loss in hyperglycemic/diabetic animals, suggesting a synergistic interaction between diabetes and the development of sAD [26]. Our data indicate a time-dependent increase in AChE (Figure 3A) and a decrease in Na^+^/K^+^-ATPase (Figure 3B) activity in the hippocampal synaptosomes following impaired insulin signaling (IP- and ICV-STZ models). These changes correlated with the cognitive decline in STZ-treated rats. Hyperglycemia modified hippocampal synaptic proteins and reduced synaptic contacts [87,88]. Impaired insulin signaling resulted in increased AChE (reduced availability of Ach) [10,11,94] and reduced Na^+^/K^+^-ATPase activity in the cerebral cortex [11,24,93], which progressed over time.

Cognitive function is strongly associated with insulin signaling pathways that regulate cell survival and synaptic dynamics/plasticity. In this study, we report a significant loss in rats’ cognition following STZ administration. This could be due to impaired insulin signaling at the receptor [95] or post-receptor level. Insulin receptors’ (IRs) expression and/or function, and Akt phosphorylation, an important regulator of learning and memory, are decreased in synaptic membranes [13,31]. Hyperglycemic rats expressed reduced levels of PI3K, Akt, and GSK-3β phosphorylation and increased levels of tau phosphorylation [20,21]. The activation (phosphorylation) of PI3K induces the activation of Akt and facilitates the inhibition (phosphorylation) of GSK-3β, a key mediator of inflammation, oxidative stress, and apoptosis [20]. Three Akt isoforms that mediate synaptic plasticity are expressed in the brain [96]. Hyperglycemia reduced the expression of IRs [95]; downregulated mediators of the insulin signaling pathway, including p-PI3K, p-GSK-3α/β, and p-Akt [31], in the cerebral cortex; and contributed to the loss of cognitive function. The early-time-course analysis in this study showed significant alterations in the levels of these signaling proteins in hippocampal synaptosomes over time, following IP- and ICV-STZ administration (Figure 4, Figure 5, Figure 6, Figure 7, Figure 8, Figure 9 and Figure 10). The effect of time post-STZ administration in most of the tested variables (phosphorylated and total proteins) was observed at 3W and 6W. However, significant changes were observed in the ratios of phosphorylated/total protein (p-Akt/Akt, p-GSK-3β/GSK-3β, p-LIMK-1/LIMK-1, and p-cofilin-1/cofilin-1) (Figure 6, Figure 7, Figure 9 and Figure 10) starting at 1W that continued to change over time (6W). In addition, our results demonstrated a profound effect of the ICV route over the IP route on the expression levels and phosphorylation states of PI3K, Akt, and PAK, perhaps due to its local impact, or their expression may be different in these two conditions (IP-STZ and ICV-STZ injection) (Figure 5, Figure 6 and Figure 8).

Synaptic dysfunction (loss of synaptic dynamics/plasticity and dendritic spine density) occurs prior to neuronal death in the brains of AD subjects. The preservation of synaptic dynamics/plasticity is partly mediated by PAK. PAK simultaneously inhibits serving and promotes F-actin branching. Changes in PAK signaling result in impaired synaptic plasticity dysfunction, and the hyperphosphorylation of tau [97] has been strongly associated with the progression of AD [43,44]. Various isoforms of PAK (PAK1-6), a family of p-21 activated kinases, are known to be linked to the phosphorylation of LIMK-1/2 for the modulation of cytoskeletal dynamics, the branching and stabilization of F-actin, and dendritic spine density. The pivotal role of PAK1/2/3 (αβγPAK) in the LIMK/cofilin pathway was recently reported to inhibit cofilin-serving activities via LIMK-1 [97]. This supports our hypothesis that impaired insulin signaling activates the αβγPAK (Figure 8) cascade and is associated with cognitive disabilities. Dendritic spines are enriched with actin cytoskeletons that regulate spine/synapse morphology, plasticity, and stability [98]. Impaired αβγPAK signaling results in the disruption of the synaptic actin-regulating machinery, LIMK-1/cofilin-1 (Figure 9 and Figure 10). Cofilin-1 is a major F-actin-depolarizing factor in neurons and is regulated by phosphorylation (inactivation). Inactive cofilin-1 (p-cofilin-1) contributes to the modification of spine morphology and synaptic dynamics/plasticity [99,100]. Cofilin-1 is phosphorylated through p-LIMK-1 or Rho-associated protein kinase (ROCK), which also leads to increased cofilin-1 activity by decreasing functional F-actin, resulting in synaptic collapse [101,102]. The dephosphorylation of cofilin-1 is facilitated by the activation of phosphatase slingshot (SSH1) [40,41,42]. Together, cofilin-1 and other actin-binding proteins regulate synaptic dynamics/plasticity, dendritic spine formation/function, neurotransmission, and mechanisms involved in learning/memory [47,48,49]. Because cofilin-1 exhibits a bidirectional effect on F-actin, depending on its relative amount, the pathological view of cofilin-1 remains debatable. The low availability of active cofilin promotes F-actin disassembly by cutting actin filaments or removing actin monomers (depolymerization), while the high availability of cofilin promotes the stabilization and nucleation of actin filaments. Thus, cofilin-1 plays a pivotal role in F-actin assembly and spine growth and maintains synaptic dynamics/plasticity [103,104,105]. Coflin-1 accumulates within senile plaques in the brains of AD patients and in experimental models of the disease [63]. Active cofilin-1 forms actin–cofilin rods and blockades of axonal traffic and affects synaptic plasticity [103,104]. Recently, p-cofilin-1 was reported to be a major factor mediating actin depolymerization and the morphological modification of dendritic spines [105], as reviewed in [106,107]. Dysregulation of p-cofilin-1/cofilin-1 levels in neurons occurred not only in AD, but also with subsequent dementia in other neurological disorders and normal aging [50,51,52].

**Figure 15:**
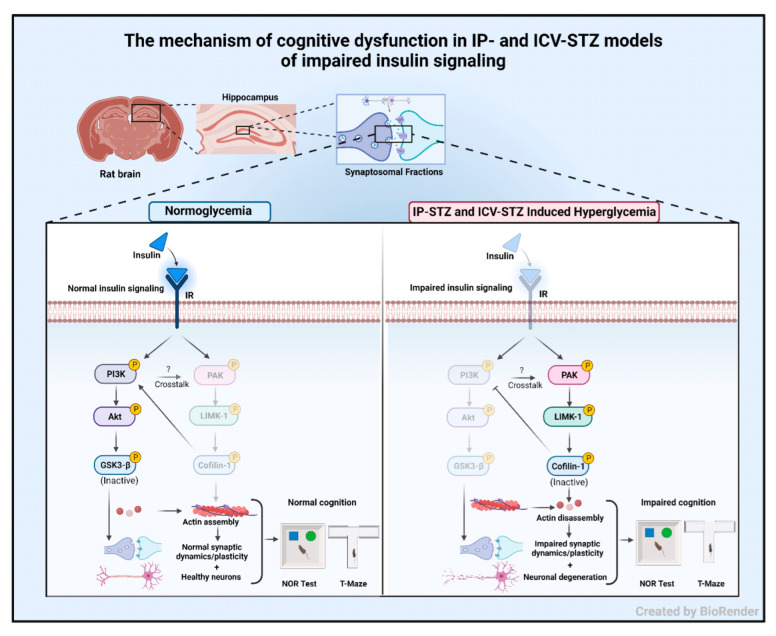
The mechanism of cognitive dysfunction in IP- and ICV-STZ models of impaired insulin signaling. Under normal conditions, insulin signaling via the insulin receptor (IR) activates PI3K/Akt signaling and inhibits GSK-3β. In addition, insulin signaling attenuates PAK/LIMK-1 signaling, leading to the activation of cofilin-1. Together, this results in improved neuronal survival and synaptic dynamics/plasticity and correlates with normal working memory and interest in new objects. Impaired insulin signaling induced by IP or ICV injections of STZ attenuates PI3K/Akt signaling and enhances GSK-3β activation. Moreover, impaired insulin signaling leads to the activation of the PAK/LIMK-1 arm and inhibition of cofilin-1. These molecular changes have been linked to impaired actin assembly and deteriorated synaptic function. These changes correlate with cognitive deterioration in STZ-treated rats and may explain early synaptic changes in sAD.

In the early stages of the disease, the causative factor for the loss of cognition is the decline/disassembly of synaptosomal F-actin [47,48,49], which was inversely correlated with the AD pathology [53,108]. An increased p-cofilin-1/cofilin-1 ratio perturbs dendritic cytoskeletal function and synergizes disease progression via synaptic energy. This augments deficits in hippocampal LTP [109,110] and leads to physiological and neurobehavioral losses [111,112]. Moreover, an imbalanced p-cofilin-1/cofilin-1 ratio leads to the displacement of drebrin from its actin-binding site and impairs dendritic spines/synapses [43]. Earlier studies demonstrated a marked decrease in dendritic spines and increased p-LIMK-1 and p-cofilin-1 in the cerebral cortex in IP-STZ [62] and ICV-STZ models [64] following oxidative stress and inflammation in the brain. The results of the current study do not only extend the findings of our previous study [3], but also support other studies that have demonstrated a role of oxidative stress [113], inflammation [114], and mitochondrial dysfunction in mediating the loss of hippocampal synaptosomes [63,66]. The PAK/LIMK/cofilin pathway is modulated by cofilin-1 binding to different partners or reactive oxygen species produced in the brain [107]. The physiological balance of nodal elements in the pathway is altered with the synaptotoxicity of AD [105] and hyperglycemia/diabetes. Our time course study implicates early changes in the PI3K/Akt/GSK-3β (Figure 5, Figure 6 and Figure 7) and PAK/LIMK-1/coflin-1 (Figure 8, Figure 9 and Figure 10) pathways in response to impaired insulin signaling. Interestingly, these changes correlated significantly with deteriorated cognitive performance (Figure 11, Figure 12, Figure 13 and Figure 14). Our data suggest that insulin signaling is essential in maintaining brain pathophysiology and cognitive function (Figure 15).

## 5. Conclusions

Our data indicate elevated AChE and declined Na^+^/K^+^-ATPase activity in hippocampal synaptosomes in response to impaired insulin signaling. Local and systemic STZ administration disrupted the balance between active and inactive nodal mediators of neuronal survival and synaptic dynamics/plasticity. The early onset of these alterations and the finding that they correlate with rats’ cognitive performance highlights the primary role of impaired insulin signaling in the onset of dementia. The present study provides mechanistic insights into the role of early changes in synaptic dynamics/plasticity in cognitive dysfunction induced by impaired insulin signaling.

## Data Availability

Available for the editorial board of the journal, if required for the publication process, but not publicly to the readers.

## Abbreviations

AD, Alzheimer’s disease; fAD, familial Alzheimer’s disease; sAD, sporadic Alzheimer’s disease; STZ, streptozotocin; IP, intraperitoneal; ICV, intracerebroventricular; ATP, adenosine triphosphate; Na^+^/K^+^-APTase, sodium–potassium adenosine triphosphatase; ACh, acetylcholine; AChE, acetylcholine esterase; ROS/RNS, reactive oxygen/nitrogen species; CNS, central nervous system; PNS, peripheral nervous system; IRs, insulin receptors; AMPA, α-amino-3-hydroxy-5-methyl-4-isoxazolepropionic acid; GluR1, glutamate receptor-1; GABA, gamma aminobutyric acid; LTP, long-term potentiation; PI3K, phosphatidylinositol-3 kinase; Akt, protein kinase B; GSK-3β, glycogen synthase kinase; PAK, p21-activated kinase; LIMK, Lin-11/Isl-1/Mec-3 kinase; ADF, actin-depolarizing factor; DTNB, 5,5-dithiobis(2-nitrobenzoic acid); BCIP/NBT, 5-bromo-4-chloro-3-indolyl-phosphate/nitro blue tetrazolium.
